# Immune cell uptake of glycinated nanoparticles conjugated to anti-fibrotic peptides enables their prolonged activity and oral administration

**DOI:** 10.1186/s12929-025-01198-8

**Published:** 2025-12-12

**Authors:** Deidree V. N. Somanader-Livera, Chen Wei, Chao Wang, Yifang Li, Dorota Ferens, Ekaterina Salimova, Cordelia Selomulya, Mohammed Akhter Hossain, Chrishan S. Samuel, Amlan Chakraborty

**Affiliations:** 1 Cardiovascular Disease Program, Biomedicine Discovery Institute and Department of Pharmacology, Monash University, Clayton, VIC 3800 Australia; 2 Monash Biomedical Imaging, Monash University, Clayton, VIC 3800 Australia; 3 School of Chemical Engineering, University of New South Wales, Sydney, NSW 2052 Australia; 4 Florey Institute of Neuroscience and Mental Health and Florey Department of Neuroscience and Mental Health, Parkville, VIC Australia; 5 School of Chemistry, The University of Melbourne, Parkville, VIC Australia; 6 Division of Immunology, Immunity to Infection and Respiratory Medicine, School of Biological Sciences, The University of Manchester, Manchester, UK

**Keywords:** Fibrosis, Cardiomyopathy, Nanotechnology, SPIONs, Relaxin, B7-33, RXFP1

## Abstract

**Background:**

Fibrosis is a hallmark of various chronic diseases, for which there is no effective cure. Whilst the recombinant form of the human peptide hormone, relaxin (RLX), is being clinically evaluated for its cardioprotective including anti-fibrotic effects in heart failure patients, this is as an injectable which is invasive. This study therefore used biodegradable nanoparticles as a delivery platform to facilitate the prolonged activity and oral application of RLX and a related mimetic as therapeutics.

**Methods:**

RLX was conjugated to glycine-functionalised biodegradable superparamagnetic iron oxide nanoparticles (SPION-RLX), enabling therapeutic levels of RLX to be systemically or orally delivered to a murine model of cardiomyopathy. The oral (p.o) application of SPION-RLX was evaluated via daily drinking water (125 ng/5mls/day) from days 7–14 or via oral gavage every 72 h (25 ng/day) from days 14–42 post-injury. The longer-term anti-fibrotic effects of p.o administered SPION-RLX (25 ng/day) or SPION-B7-33 (25 ng/day), a single-chain RLX derivative and relaxin family peptide receptor 1 (RXFP1) agonist were compared to the frontline ACE inhibitor, perindopril (60 ng/day) from days 14–42 post-injury.

**Results:**

SPION-RLX was likely phagocytosed by surveiling RXFP1-expressing dendritic cells (DCs) and transported to the circulation and target site. This allowed for the systemic or oral administration of SPION-RLX to maintain its anti-fibrotic efficacy in mice with cardiomyopathy and restore organ dysfunction after 7 days of treatment. Single-cell transcriptomics provided insights into the phagosomal uptake of SPION-RLX which may have been mediated via scavenger receptors expressed by DCs. When orally administered every 72 h to mice with established cardiomyopathy over a 4 week period, SPION-RLX or SPION-B7-33 demonstrated greater anti-fibrotic efficacy than perindopril.

**Conclusion:**

The conjugation of RXFP1-binding peptides to glycine-functionalised biodegradable SPIONs allowed for their circumnavigation of the gut, and prolonged activity as orally administered therapies. These findings have significant ramifications for the oral administration of peptide therapies in general.

**Supplementary Information:**

The online version contains supplementary material available at 10.1186/s12929-025-01198-8.

## Background

Peptide therapies have been recognised for being highly selective, efficacious [1, 2], and well tolerated in human patients, highlighting their great potential as therapeutics. However, the clinical application of peptide drugs is limited by their short half-lives and plasma stability (as they are broken down by circulating and tissue resident proteases) and poor absorption through the gut [2, 3]. Thus, the majority of peptide therapeutics, such as insulin, glucagon-like peptide- 1 and somatostatin analogues, need to be systemically administered via daily injection(s) or continuous infusion to maintain their activity, which are both invasive and cumbersome. Hence, new technologies or drug delivery vehicles that can improve the oral applicability of peptides would revolutionise their application as medicines.

Superparamagnetic iron oxide nanoparticles (SPIONs) are FDA approved for the magnetic resonance imaging of the lung and various cancers [4], and were intentionally designed to degrade within 48–72 h, to avoid any cytotoxic effects from accumulating in the body [5, 6]. We have found that SPIONs can be coated or functionalized with the non-essential, immunomodulatory amino acid, glycine, to improve their conjugation to various compounds and render them as non-cytotoxic, biocompatible drug delivery vehicles [7–9]. Additionally, glycine-coated SPIONs with a hydrodynamic size < 100 nm demonstrated immunomodulatory properties by dampening the host inflammatory response upon their administration [8, 10], and could be phagocytosed by infiltrating and resident antigen presenting cells (such as dendritic cells and macrophages) [9, 11]. These glycine-functionalised SPIONs can migrate to injured (target) sites via immune cell chemotaxis, whereby compounds conjugated to them and released upon their degradation could exert therapeutic effects in a targeted manner without eliciting an immune response [9, 10].

In a recent study, we conjugated glycine-coated SPIONs to the short-acting and gut-impermeable anti-fibrotic peptide drug, serelaxin [12] (recombinant human gene-2 relaxin; RLX [13, 14]), which is being clinically evaluated by various pharmaceutical companies as an injectable treatment for patients with heart failure [15]. When delivered intranasally (i.n) to a murine model of chronic allergic airways disease, SPION-RLX was taken up by infiltrating and tissue-resident macrophages, which enabled the conjugated RLX (which has an in vivo half-life of ~ 4–8 h [16, 17]) to be preserved for at least 48–72 h before the degradation of SPIONs resulted in the release of RLX into the airways/lungs of mice [9]. Strikingly, this allowed for i.n-administered SPION-RLX to markedly attenuate several features of airway/lung inflammation, remodelling and hyperreactivity (breathing dysfunction) to the same extent as continuous minipump-infused RLX after a 7-day treatment-period [9].

As the size-dependent, glycine functionalisation-mediated uptake of SPION-RLX by egressing and tissue-resident immune cells potentially provided a resource by which RLX could bypass the gut, in this study we determined if the therapeutic effects of SPION-RLX could be maintained when systemically- or orally-administered to a preclinical model of cardiomyopathy. We determined the immune cell subsets that were taking up and delivering SPION-RLX to the appropriate site of injury in the model of cardiomyopathy and in a lipopolysaccharide (LPS)-induced acute lung inflammation model. The longer-term therapeutic effects of orally-administered SPION-RLX or SPION-B7-33, a single-chain RLX-derivative which activates relaxin family peptide receptor 1 (RXFP1) at the same site as RLX [18], was also evaluated in comparison to the orally-delivered angiotensin-converting enzyme inhibitor, perindopril, in mice with established cardiomyopathy and LV fibrosis. Single-cell transcriptomics analysis of a scRNAseq dataset from the murine heart was used to provide mechanistic insight into the findings acquired.

## Materials and methods

### Materials

Isoprenaline hydrochloride (ISO; I5627) and methacholine (A2251) were obtained from Merck/Sigma-Aldrich (St. Louis, MO, USA). Lipolysaccharide (LPS; tlrl-eklps) was purchased from InvivoGen (San Diego, CA, USA). Recombinant H2 relaxin (serelaxin; RLX) was kindly provided by Corthera Inc. (San Mateo, CA, USA; a subsidiary of Novartis International AG; Switzerland). Perindopril was purchased from MedChemExpress (Monmouth Junction, NJ, USA).

### Synthesis and pharmacokinetics of SPION-conjugated RLX (SPION-RLX), FITC-labelled SPION-RLX (SPION-RLX.^FITC^) and SPION-conjugated B7-33 (SPION-B7-33)

Carboxyl-coated SPIONs were synthesized utilizing the modified alkaline co-precipitation method used previously [8, 9]. SPIONs were then conjugated to the N-terminus of RLX (at a concentration of 0.05 mg/ml) or B7-33 (0.05 mg/ml) using carbodiimide chemistry as described previously [9] (schematically shown in Additional file 1: Fig. S1). The pharmacokinetics of SPION-RLX had been evaluated previously [9], where it was found that 0.05 mg/ml RLX conjugated to SPIONs resulted in ~ 10.5 µg of RLX being conjugated. To a subset of SPION- conjugated RLX, fluorescein isothiocyanate (FITC) was conjugated to lysine residues on RLX as before [9]. SPION-RLX and SPION-RLX^FITC^ conjugated nanoparticles were characterized for their size, hydrodynamic size, stability and charge to validate the conjugation. Briefly, the size of the particles was measured using a QUANTAX analysis system (Bruker, Billerica, MA, USA) and imaged using a FEI Tecnai G2 F20 S-TWIN field emission gun transmission electron microscope (FEI Company, Hillsboro, OR, USA) connected to a wide-angle Orius SCD200 CCD camera, and was evaluated to have a core diameter of 26.28 nm, which extended to 41.20 nm following the conjugation of RLX (shown in Additional file 1: Fig. S2A). The hydrodynamic size was measured using a Malvern Nano-Zetasizer (Malvern Panalytical, Malvern, Worcestershire, UK) along with the poly-dispersity index (pDI) for stability of the SPION-RLX, which were 88.5 ± 21 nm and 0.210, respectively (shown in Additional file 1: Fig. S2B), indicating good colloidal stability. RLX^FITC^ conjugation to the nanoparticles was validated using a fourier-transformed infrared radiation (FTIR) spectrophotometer (Agilent Technologies, Santa Clara, CA, USA) with a pointed probe which demonstrated the presence of NH_2_ (amino acid group) at 3248 cm^−1^ and carbonyl group of lactone ring in FITC at 1723 cm^−1^ as well as (C-N) stretching at 1090 cm^−1^ (shown in Additional file 1: Fig. S2C). Conjugation of RLX to SPIONs (showed in red) and RLX^FITC^ to SPIONs was validated by the charge of the particles (zeta-potential) calculated as − 2.75 mV and − 7.45 mV, respectively (shown in Additional file 1: Fig. S2D).

This SPION-conjugated RLX was then diluted to produce 125 ng in 5 ml (25 ng/ml) of drinking water, for the oral consumption of SPION-RLX via drinking water; or 25 ng per 200 µl of water (125 ng/ml) for the intraperitoneal (i.p) or oral gavage (p.o) administration of SPION-RLX. As B7-33 mimics the cardioprotective effects of RLX at an equimolar dose [18] or equivalent dose corrected for molecular weight [18, 19], SPION-B7-33 was also evaluated at 25 ng per 200 µl of water, when oral gavage administered. Moreover, the maximum tolerant dose of SPIONs has been previously verified, where the cell viability of human A549 cells remained at 100% following treatment with up to 400 µg/ml of SPIONs [8]. However, this study only utilized a SPION load of 200 µg/ml for the conjugation of SPION-RLX or SPION-B7-33, that was used for the animal studies outlined. For the studies involving the LPS model, SPION-RLX^FITC^ (with a SPION load of 200 µg/ml) was used to study the uptake of RLX by immune cells subsets.

### Experimental design

11–12-week-old male C57BL/6J mice, weighing 26–30 g were obtained from the Monash Animal Research Platform (MARP; Monash University, Clayton, Victoria, Australia) and used to establish and treat the model of cardiomyopathy outlined below. Male mice were used as they are more susceptible to develop cardiomyopathy-induced heart failure (HF) [20, 21]. To determine the immune cell subsets that were taking up the SPION-RLX, a model of acute lung injury was also established in 6–8-week-old female Balb/c mice (provided by MARP), given that female Balb/c mice are more susceptible to developing airway inflammation compared to their male counterparts [22]. In each case, mice were housed under standard conditions in the mouse facility of the Department of Pharmacology, with ad-libitum access to a standard chow diet (Barastock Stockfeeds; Pakenham, Victoria, Australia) and water, on a 12-h light/12-h dark cycle. Mice were allowed to acclimatize for 6–7 days prior to being subjected to any experimental procedures. All experiments were approved by Monash University’s Animal ethics Committee (under MARP/2020/26910 or MARP/2021/29157) in line with the Australian Code of Practise for the Care and Use of Laboratory Animals for Scientific Purposes. Animals were randomized via a blinded analysis into groups of equal sizes: n = 7–8 for the cardiomyopathy model and n = 6 for the acute lung injury model. Power calculations were carried out to ensure that with a 25% standard deviation, the study would be 80% powered to detect a 20–30% effect, with n = 6–8 animals per group, respectively.

### Establishment and treatment of the isoprenaline-induced model of cardiomyopathy

The isoprenaline (ISO)-induced murine model of dilated cardiomyopathy was utilised in this study [19, 23]. Groups of mice (n = 8/group) were subjected to once daily subcutaneous administrations of ISO (25 mg/kg body weight (BW)) for five consecutive days. Mice were then left untreated for a further 9 days (until day 14) for fibrotic healing to occur (injury control/ISO group). ISO is a synthetic catecholamine and β-adrenoceptor agonist that stimulates a positive chronotropic effect in the heart. Hence, when repeatedly administered over 5 days, the heart undergoes an aberrant wound healing-induced fibrosis owing to its limited capacity to heal. This eventually resulted in LV remodelling and dysfunction by day 14 [19, 23]. A separate group of mice (n = 8), that were injected once daily with saline the vehicle for ISO) for five consecutive days and left untreated until day 14 were included as a non-injury control group.

In addition to the two control groups established, four separate sub-groups of ISO-injured mice received various treatments from days 7 to 14 post-injury (n = 8/group). One sub-group was subjected to the subcutaneous implantation of osmotic minipumps (model 1007D) containing RLX (0.5 mg/kg/day; which continuously infused RLX into the circulation of mice, at a dose that was bioactive in mice [19, 23–25]. This dose of minipump infused RLX (0.5 mg/kg/day) was shown to produce circulating H2 relaxin levels of ~ 15–25 ng/ml after 5–7 days post-infusion [26], which was equivalent to the supraphysiological RLX levels found in pregnant women producing twin or triplet foetuses at the same time [27]. Two separate sub-groups of ISO-injured mice were either intraperitoneally (i.p) administered with 25 ng of SPION-RLX (in 200 µl of water) on days 7, 10 and 13 post-injury (ISO + SPION-RLX (i.p) group); or was given 125 ng of SPION-RLX in 5 ml (25 ng/ml; in 15 ml Falcon tubes with stoppers) of drinking water each day over the 7 day treatment period (ISO + SPION-RLX (p.o) group). To ensure that all mice drank a similar volume of water containing SPION-RLX, these mice were individually housed. Another separate subgroup of ISO-injured mice was also administered with empty nanoparticles (ISO + Empty-SPIONs) that were not conjugated to RLX, either i.p (n = 4) or p.o (n = 4). All mice underwent systolic blood pressure (SBP) measurements on days 0, 7 and 14 post-injury. Furthermore, all mice with the exception of ISO + Empty-SPION group, also underwent transthoracic echocardiography on day 14. Mice were then humanely killed by cardiac puncture on day 14, for blood (plasma) and tissue collection.

In a separate study, a 42 day ISO-induced model of cardiomyopathy was later utilised to evaluate the longer term (4 week) effects of p.o-administered SPION-RLX (25 ng/day) or SPION-B7-33 (25 ng/day) in comparison to that of p.o-delivered (unconjugated) RLX (25 ng/day) or B7-33 (25 ng/day) alone, Empty-SPIONs alone or the clinically-used ACE inhibitor, perindopril (60 ng/day; equivalent to ~ 1–2 mg/kg/day; a dose previously demonstrated to induce anti-hypertensive and anti-fibrotic effects in ISO-injured mice [19]), (n = 7/group) when administered from days 14 to 42 (weeks 2 to 6) post-injury. The saline and ISO alone controls (n = 7/group) were established as outlined for the 14 day model, then maintained until day 42 post-injury. Sub-groups of ISO-injured mice were administered with each of the above-mentioned treatments via oral gavage, every 72 h from day 14 until day 42. Only the saline and ISO alone controls, and the ISO + perindopril-treated group underwent SBP measurements at day 14 (prior to perindopril treatment) and at day 42 (after 4-weeks of perindopril treatment)—to confirm that the ACE inhibitor was active at the dose administered. Mice were then humanely killed by cardiac puncture on day 42, for blood and tissue collection.

The safety of repeated Empty (glycine functionalised)-SPION administration was also separately determined over a 6 week period. Healthy male (n = 12) and female (n = 12) mice were either left untreated for 6 weeks (n = 6/sex) or oral gavage treated with Empty-SPIONs every 72 h (15 times) over the 6 week period (n = 6/sex). At the completion of this study, mice were sent to an independent pathology company (Cerberus Sciences, Scoresby, Victoria, Australia) to have their major organs and glands histopathologically assessed.

### Establishment and treatment of the lipopolysaccharide-induced model of acute lung injury

To determine which subset of immune cells were taking up SPION-RLX and whether these cells expressed the cognate receptor, RXFP1, a lipopolysaccharide (LPS)-induced model of acute lung injury (ALI) was established in 6–8-week-old female BALB/c mice (n = 6/group). On day 0, mice were briefly anaesthetised via isoflurane (Aerrane; 2–3% in oxygen; Baxter Healthcare; Toongabbie, New South Wales, Australia) then sensitised with i.n administered LPS (50 μl of a 5 μg/ml solution; 250 ng/mouse). LPS was administered intranasally to directly induce lung damage, influx of pro-inflammatory cells and emphysema. One sub-group of LPS-sensitized mice (injury control/LPS group) were left untreated until day-7 (a time-point at which they developed significant lung inflammation and related AHR [28, 29]). A separate sub-group of LPS-instilled mice (n = 6) were randomly allocated to receive i.n administration of SPION-RLX^FITC^ (25 ng/day; LPS + SPION-RLX^FITC^ group), every 48 h on days-2, -4 and -6 post-LPS-induced lung injury. An uninjured control group of mice (n = 6) were administered 50 μl of saline (SAL) intranasally on day 0 instead of LPS, and were left untreated until day 7.

### Subcutaneous implantation of osmotic minipumps

Mice were initially anaesthetized with isoflurane (2–3% in oxygen) using an anaesthetic induction chamber. Once anaesthetized, mice were maintained in a supine position and an 8-10 mm incision was then made between the scapulae, so that a skin pocket could be created with blunt scissors. The minipump (model 1007D, Alzet®; Cupertino, CA, USA; with had an infusion rate of 0.5 µl/hour for 7 days) was then implanted into the skin pocket with the open end of the pump facing the tail of the mouse. The incision site was then closed with Michel clips and mice were monitored until they regained consciousness.

### Oral gavage administration of treatments

At the appropriate time, mice were picked up using the scruff hold and a 22-gauge curved gavage needle connected to 38 mm tubing was inserted into the mouth of each mouse, to administer the appropriate treatment at a volume of 200 µl per administration.

### Measurement of blood pressure using tail cuff plethysmography

To investigate the effects of ISO-induced cardiomyopathy and the subsequent treatments evaluated on SBP, tail cuff plethysmography (MC4000 Blood Pressure System; Hatteras Instruments Inc.; Grantsboro, NC, USA) was conducted on days 0, 7 (prior to treatment) and 14 (7 days post-treatment) of the short-term model or days 14 (prior to treatment) and 42 (28 days post-treatment) of the longer-term model. In line with previous studies, 15–20 SBP measurements were obtained to achieve a pooled mean for each animal [19, 23, 24].

### Evaluation of LV function using transthoracic echocardiography

To elucidate the effects of ISO and the subsequent treatments evaluated on LV stiffness and function, transthoracic echocardiography was performed using the Vevo 2100 Imaging system (housed at the Monash Biomedical Imaging Facility; Clayton, Victoria, Australia), on all groups of mice (with the exception of the ISO + Empty-SPION group) at day 14 post-saline or ISO administration [23]. Echocardiographic measurements were obtained from grey-scale two-dimensional (2D) B-mode images acquired in the parasternal long-axis view and M-mode images at the midpapillary level in the parasternal short-axis view. The animal was then tilted backwards in the Trendelenburg position to obtain the four-chamber view through the apex of the heart to perform pulsed-wave Doppler imaging.

### Tissue, plasma and bronchoalveolar lavage fluid (BALF) isolation

On day 14 or day 42 post-saline or ISO administration, all mice were initially weighed and subsequently killed via cardiac puncture following an overdose of isoflurane (5% in oxygen). Approximately ~ 500-700µL of blood was withdrawn and placed into a heparinised tube (Minicollect Greiner Bio-One; Kremsmünster, Austria). Heparinised tubes were centrifuged at 4ºC for 10 min at 12,000 rpm for the isolation and collection of plasma, which was stored at -80ºC until required for the quantification of circulating RLX levels. Following this, the heart was then isolated, blot-dried and weighed (heart weight (HW)), and the atria and right ventricle were trimmed off to isolate the LV. The LV was then separately weighed (LV weight (LVW)) and transversely sectioned into the apex, mid-zone, and base. The apex was repeatedly washed in Dulbecco’s phosphate-buffered saline (dPBS) to remove any blood cells in preparation for flow cytometry analysis. The base was snap frozen in liquid nitrogen and stored at -80ºC until required for protein extraction analyses. The mid-zone was fixed in 10% neutral buffered formalin (NBF) to be processed for histological and immunohistochemical (IHC) analyses. For the groups treated with SPIONs, the liver was also isolated and fixed in 10% NBF in a cassette. Both the mid-zone and liver sections were then sent to Monash Histology Platforms for tissue processing.

On day 7 post-saline or LPS administration, bronchoalveolar lavage fluid (BALF) was collected from each anaesthetised mouse with three repeated washes (400 μl each). The BALF was spun in a centrifuge for 4 min at 4 ºC at 1500 rpm to isolate cells. Each supernatant was removed and was subject to a protein assay. BALF cells were treated with ACK (ammonium and potassium citrate) buffer to lyse red blood cells, then resuspended in FACS buffer and stored on ice for future cell counting. Mice were then culled for lung tissue removal, which was weighed, and separated into four separate lobes. The largest lobe was fixed in 10% NBF overnight and subsequently sent to Monash Histology Platform to be processed, embedded in paraffin wax, and sectioned for analysis by tissue histopathology. The second largest lobe was prepared for FACS analysis, whilst the remaining two lobes were snap-frozen in liquid nitrogen and stored at − 80 °C.

### Flow cytometry analysis of immune cell influx

To elucidate the effects of ISO administration and the subsequent treatments evaluated on immune cell populations within the LV, populations of regulatory T cells (Tregs), M2-like macrophages and dendritic cells (DCs) were quantified by flow cytometry, as described before [9]. To elucidate the effects of LPS administration and the uptake of SPION-RLX^FITC^ administration within the airways/lung, populations of DCs were quantified. The cells from the LV apex or second largest lung lobe were isolated and resuspended in a FACS buffer (dPBS + 5% FCS + 0.5 mM EDTA), from which FACS sorting was carried out on 1 × 10^5^ cells/ml. Initially, samples were incubated with a rat anti-mouse CD16/32 Fc block (#553141; 1:100 dilution; BD Horizon; Franklin Lakes, NJ, USA) to prevent non-specific Fc binding. Cells were then stained with fluorescently-labelled primary antibodies, as detailed below.

**Table:** 

Antibody	Cat. No	Source	Dilution used
CD45-PE-Cy5 (anti-mouse)	553,082	BD Biosciences, San Jose, CA, USA	1:200
CD4-BUV496 (anti-mouse)	741,050	BD Biosciences, San Jose, CA, USA	1:200
CD25-BV785 (anti-mouse)	564,368	BD Biosciences, San Jose, CA, USA	1:100
Foxp3-V450-BV421 (anti-mouse)	561,293	BD Biosciences, San Jose, CA, USA	1:100
F4/80-APC-Fire750 (anti mouse)	123,151	BioLegend, San Diego, CA, USA	1:100
CD206-AF647 (anti mouse)	141,711	BioLegend, San Diego, CA, USA	1:100
CD11c-BUV395 (anti mouse)	564,080	BD Horizon, Franklin Lakes, NJ, USA	1:100

Another set of cells from a saline-treated mouse were left unstained to act as the unstained control. For gating specific cell subsets, three fluorescent minus one controls (FMOs) were prepared. Subsets of cells were either stained with 1) all primary antibodies except FoxP3 (FMO1); 2) all antibodies except CD45 (FMO2); or 3) all antibodies except for CD206 (FMO3). All cells were washed in FACS buffer before proceeding to live/dead staining. Subsequently, all cellular subsets except the unstained control and FMOs were subjected to secondary staining with Zombie aqua dye (#423101; 1:1000 dilution; BioLegend; San Diego, CA, USA) for live/dead screening. For intracellular FoxP3 staining, all cells were permeabilised with a permeabilization buffer (dPBS + 1% Triton-X) and incubated in the dark for 15 min. The cells were washed and resuspended in dPBS containing 0.1% Triton-X followed by the addition of FoxP3 primary antibody (prepared in permeabilization buffer). Cells were subsequently incubated in the dark for 20 min and were washed in FACS buffer, before re-fixing occurred in 1% paraformaldehyde. Cells were acquired on a BD Fortessa X-20 Cytometer (FlowCore Platform; Monash University, Clayton, Victoria, Australia). Data were analysed using Flow Jo™ v10.8 (BD Biosciences; San Jose, CA, USA). Initially, gating was performed as FSC-A and SSC-A, followed by FSC-H and FSC-A for doublet discrimination/exclusion. The single cells obtained were used for live/dead screening and live cells (zombie^neg^) and were used for further analysis. Using the FMOs and unstained cells, M2-like macrophages were gated as CD45^+^ cells followed by gating of F4/80^+^CD206^+^ cells. T_regs_ were gated and classified as CD4^+^CD25^+^Foxp3^+^ cells, whilst DCs were gated as CD11c^+^ cells.

### ELISA analysis

To correlate the circulating RLX levels with its therapeutic effects from the 14 day ISO model, RLX levels in the plasma of ISO-injured mice treated with Pump-RLX, SPION-RLX (i.p or p.o) or Empty-SPIONs at day 14 post-injury were quantified using the H2 RLX Quantikine ELISA kit (DRL200; R&D Systems, Minneapolis, MN, USA). The ELISA was performed according to the manufacturer’s instructions, with all standards and samples assayed in duplicates, as detailed previously [9]. To analyse LV TGF-β1 (pro-fibrotic cytokine) levels, total protein was extracted from the basal portion of the LV, from all mice that underwent the 42 day ISO model using a previously described method [30]. LV TGF-β1 expression levels were then detected in each sample using the mouse TGF-β1 DuoSet ELISA (#RDSDY167905; R&D Systems); with each sample assayed in duplicate according to manufacturer’s instructions, against serially-diluted standards.

### Histological staining and analysis

Serial 5 µm LV sections (from mice subjected to saline or ISO) were stained with H&E (to measure LV inflammation) or 0.1% picrosirius red (Polysciences Inc.; Warrington, PA, USA; to measure interstitial LV fibrosis and LV cardiomyocyte hypertrophy [19, 23, 24]). An additional serial LV section along with 5 µm liver sections from these mice were also stained with Perl’s Prussian blue staining (which can detect the iron core of NPs; to identify the distribution of SPIONs in these organs) and counterstained with neutral red [9]. A 5 µm lung section (from mice subjected to saline or LPS) were stained with H&E (to measure lung inflammation). All staining was performed by the Monash Histology Platform (Clayton, Victoria, Australia), and all stained slides were then digitally scanned using the Aperio Scanscope AT Turbo scanner (Leica Biosystems; Nußloch, Baden-Württemberg, Germany), whereby the high-resolution images were stored on a local server associated with the instrument.

The morphometric analysis of various end-points was then performed in a blinded fashion using the Aperio ImageScope v.12.4.3 software (Leica Biosystems). Given the low levels of LV inflammation detected in ISO-injured mice, the infiltration of inflammatory cells into the LV myocardium was semi-quantified from 10 random and non-overlapping fields of view (FOV) at × 200 magnification. These cell counts were then pooled to obtain the mean number of inflammatory cell counts for each animal. The picrosirius red-stained interstitial LV collagen deposition was determined from 10 random and non-overlapping FoV (at × 200 magnification) per section and expressed as a fraction (%) of the total area stained [19, 23]. To quantify cardiomyocyte hypertrophy, picrosirius red-stained LV sections were morphometrically analysed at × 400 magnification. Within each section analysed, the area of 10 random cells from 10 random nonoverlapping FOV was traced and measured, to obtain a mean cardiomyocyte area per section from 100 non-overlapping cardiomyocytes [19, 23]. The blue-stained NPs from the Perl’s Prussian blue staining were just visualised for their distribution within the LV and liver [9].

### IHC staining for markers of LV inflammation, fibrosis and angiogenesis

Immunohistochemistry was performed to quantify the expression of pro-inflammatory and profibrotic markers within the injured LV myocardium (of saline and ISO-injected mice). Separate serial mid-zone sections were stained with either a polyclonal IgG antibody to tumour necrosis factor (TNF)-α (ab6671; 1:250 dilution), interleukin (IL)-1β (ab205924; 1:500 dilution) or TGF-β1 (ab92486; 1:250 dilution; all from Abcam Antibodies; Cambridge, MA, USA); or a monoclonal IgG2A clone 1A4 antibody to α-SMA (a marker of myofibroblast differentiation and smooth muscle-associated blood vessel density; M0851; 1:1000 dilution; Agilent Technologies (Dako); Mulgrave, Victoria, Australia). The following day, the respective sections were stained with either Dako Envision^+^ System kits containing either a HRP-labelled anti-rabbit secondary antibody (K4003; for the detection of TNF-α, IL-1 β or TGF-β 1) or HRP-labelled anti-mouse secondary antibody (K4000; for the detection α-SMA). Antibody binding was visualised by 3,30-diaminobenzidine (DAB; Dako), before slides were counterstained with haematoxylin and mounted in DePex (VWR International; Radnor, PA, USA).

IHC-stained slides were also scanned using the Aperio Scanscope AT Turbo scanner and analysed in a blinded fashion using the Aperio ImageScope v.12.4.3 software. For TGF-β1 staining, the strong positive DAB (brown)-staining from 10 random and non-overlapping FoV (at × 200 magnification) per section was detected and expressed as a fraction (%) of the total area stained. For TNF-α, IL-1β and α-SMA, the number of positive DAB-stained cells per FOV were counted at magnifications of × 400, × 100 or × 200, respectively, and expressed as the number of positively-stained cells per field. Additionally, the number of α-SMA-stained blood vessel density was counted from 10 random and non-overlapping FoV (at × 100 magnification) per section, to provide a measure of vascular rarefaction in each of the groups evaluated.

### Western blotting

To assess the myocardial expression levels of matrix metalloproteinase (MMP)-13 (collagenase 3; the primary collagenase in rodents) and tissue inhibitor of metalloproteinases (TIMP)-1 and TIMP-2, equivalent amounts of LV protein (from the ISO model; 10–15 μg per sample) were run on 4–15% SDS-PAGE gels and analysed by Western blotting [23, 25]. Changes in MMP-13 and TIMP expression were assessed using a monoclonal (IgG_1_) antibody to detect MMP-13 (#MA5-14238; 1:1000 dilution; ThermoFisher Scientific; Scoresby, Victoria, Australia); a polyclonal (IgG) antibody to detect TIMP-1 (ab38978; 1:1000 dilution; Abcam Antibodies) or rabbit monoclonal (IgG) antibody to detect TIMP-2 (#5738; 1:1000 dilution; Cell Signaling Technology; Danvers, MA, USA). In each case, these membranes were reprobed with a rabbit monoclonal (IgG) antibody to detect GAPDH (Cell Signaling Technology), to confirm the equivalent loading of protein samples. In all cases, membranes were further probed with a goat anti-rabbit HRP (#7074; 1:2000 dilution; Cell Signaling Technology) or horse anti-mouse HRP (#7076; 1:2000 dilution; Cell Signaling Technology) IgG secondary antibodies, respectively. Proteins were then detected using the Clarity Western ECL substrate detection kit and quantified by densitometry with a ChemiDoc MP Imaging System and Image Lab v.6.0 software (both from Bio-Rad Laboratories, Hercules, CA, USA) [23, 25]. The densitometry values were then expressed relative to the value from the saline-treated control group, which was expressed as 1 in each case. The MMP-13 to TIMP-1 ratio from each sample evaluated was also generated. Representative blots of the appropriate end points determined were also chosen for presentation in each case.

### Gelatin zymography

Equivalent amounts of LV protein (from the ISO model; 5–10 µg per sample) were additionally analysed on SDS-PAGE gels consisting of 7.5% acrylamide and 1 mg/ml gelatin, to assess changes in MMP-2 (gelatinase A) and MMP-9 (gelatinase B) levels from all groups studied, as detailed previously [23, 30]. Gelatinases can further degrade collagenase-digested collagen into gelatin, and gelatinolytic activity was indicated by clear bands and assessed by densitometry of the relevant bands, which was also expressed relative to the value from the saline-treated control group, which was expressed as 1 in each case. The MMP-9 to TIMP-1 and MMP-2 to TIMP-2 ratio from each sample evaluated was also generated. Representative zymographs of the appropriate end points determined were also chosen for presentation in each case.

### scRNA-seq and snRNA-seq data analysis using datasets from ParseBiosciences

Raw data from a ParseBiosciences C57BL/6 mouse heart dataset was available for analysis using an open-source single cell analysis Toolkit, Trailmaker Platform (Cellenics®; https://github.com/hms-dbmi-cellenics). Quality control, integration and clustering were performed with Seurat version 4.0.1 [31]. Loading the Seurat object in Cellenics, cells or nuclei that expressed less than 200 genes were filtered out, and the genes detected in less than 3 cell clusters were removed. Cells with a percentage of UMIs mapped which were less than 20% for mitochondrial genes were retained for analysis. The SCT transformed algorithm to normalize the sample and select the top highly variable genes was then applied, to revert from effects of cell cycle and stress associated changes in expression. The resulting UMIs were screened for doublets and a Principal Component Analysis (PCA) was performed on the top 3000 variable genes and top 28 principal components, then the data was clustered based on the Principal Component Elbow Plot. Cell clusters with ~ 92% variation were visualized using Unique Manifold Approximation and Projection (UMAP) with a minimum Euclidean distance of 0.2 performing leiden clustering algorithm at a resolution of 0.2. Differential expression of genes detected in a minimum of 10% of all cells or nuclei, a minimum of 0.2 average log fold-change (FC), and a minimum of 0.05 adjusted p-value were implemented into the algorithm. The major cell types (sc-types) were automatically annotated for the heart scRNA-seq dataset using cell specific canonical gene markers from PantherDB [32]. Expression of relaxin receptors *RXFP1, RXFP2, RXFP3* and RXFP4 was evaluated in individual clusters. CellChat (v1.1.3) [33] was used to generate communication probability based on the calculation of average expression of *RXFP1* in individual clusters with all other clusters. Cell interactions with a p-value < 0.05 were termed significant. The interaction of *RXFP1* was subjected to Enrichment analysis using EnrichR for the top 100 hit genes and evaluated for stacking based on a cumulative scoring of single-sided Fisher’s exact tests, FDR represented for adjusted P value after multiple tests. Differential expression of scavenger receptors and *RXFP1* in each individual cluster was analysed to infer cell specific expression.

### Statistical analysis

All data were expressed as the mean ± standard error of the mean (SEM). Data analysis was performed using GraphPad Prism v9.1.2, from group sizes of n = 6–8 per group, where n denotes the number of independent samples obtained from each group. Changes in SBP were analysed using a two-way ANOVA (to assess the effects of treatment vs time), whilst all other endpoints were analysed using a one-way ANOVA. Post-hoc comparisons between groups were only carried out if the p-value of the overall ANOVA was statistically significant (i.e. *P* < 0.05) and there was no significant variance in homogeneity. A Bonferroni post-hoc test was applied to the two-way ANOVA, whilst a Tukey’s post-hoc test was applied to the one-way ANOVA to allow for multiple comparisons to be made between the appropriate groups. For the (MMP, TIMP and MMP to TIMP ratio) data that was normalised to the saline control group, all individual values from each group (including those from the saline control group) were normalised to the mean of the saline group, which was expressed as 1 in each case. In this case, these data were analysed using a nonparametric (Kruskal–Wallis) test and Dunn’s post-hoc test. Differences were considered statistically significant at *P* < 0.05.

## Results

### The systemic or oral delivery of glycinated SPION-RLX attenuated LV immune cell infiltration and pro-inflammatory cytokine expression in mice with cardiomyopathy

**Fig. 1:**
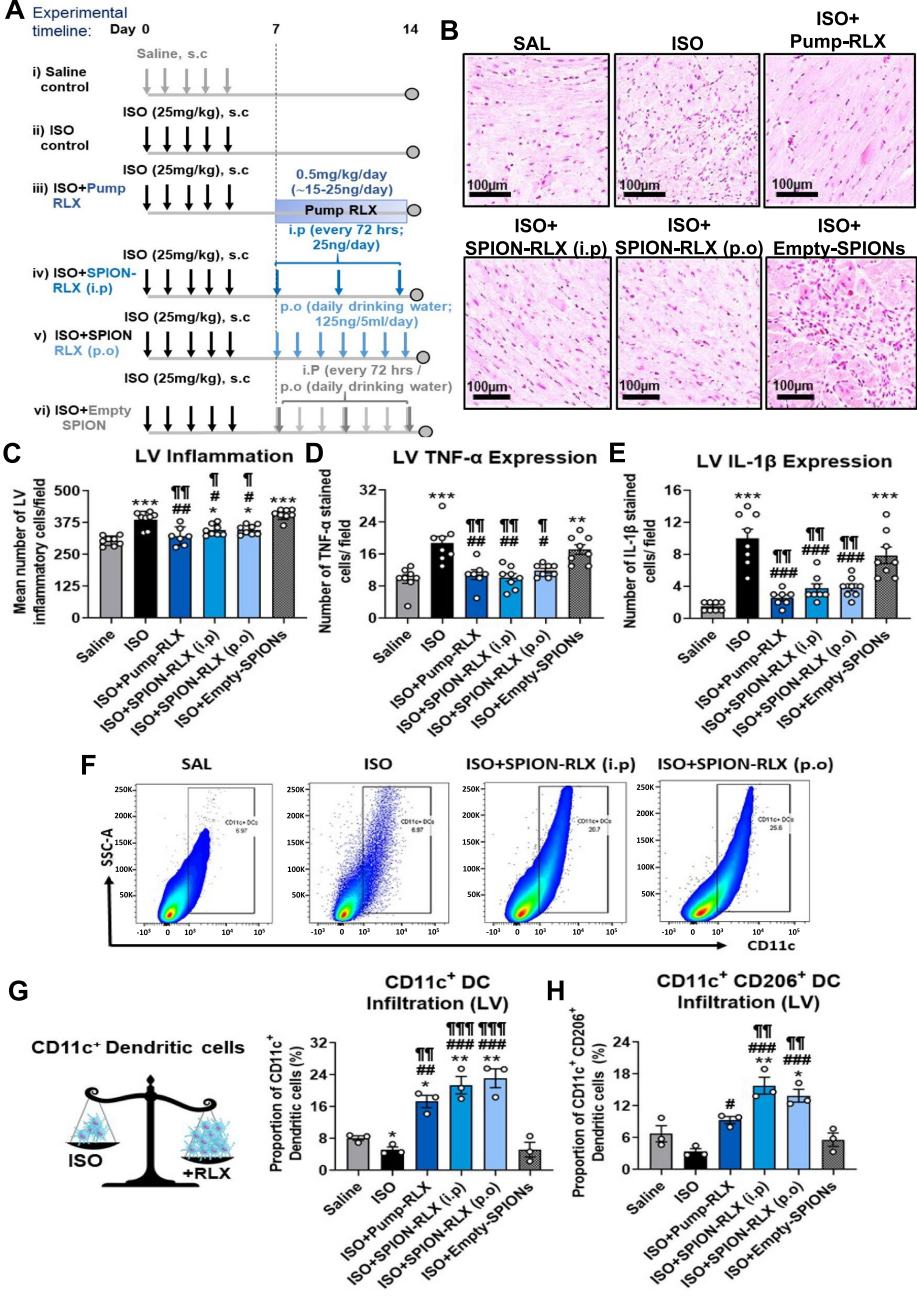
The effects of minipump (Pump)-infused RLX vs i.p- or drinking water (p.o)-delivered SPION-RLX on measures of LV inflammation in mice with cardiomyopathy, when administered from days 7–14 post-injury. **A** Schematic outline of the 14 day model of cardiomyopathy established and treatments (timing, delivery routes, dosing) evaluated. **B** Representative images of hematoxylin and eosin (H&E)-stained LV tissue sections show the extent of LV inflammatory cell infiltration in each of the groups investigated. **C–E** Also shown are the mean ± standard error of the mean (SEM) number of **C** LV inflammatory cells per field, **D** TNF-α levels and **E** IL-1β levels in each group. **F** Representative FACS plots show the extent of CD11c^+^ DC infiltration within LV tissue from each of the groups indicated. **G**, **H** Also shown are the mean ± SEM LV **G** CD11c^+^ DCs and **H** CD11c + CD206 + DCs in each of the groups investigated. The data presented in panels **C**–**E** were obtained from n = 7–8 mice per group; whereas the data from panels **G** and **H** were obtained from n = 3 separate assays (tissue pooled from n = 2–3 mice per assay; from n = 7–8 mice per group). The white coloured circles in each of the bar plots represent the individual data points per group. *p < 0.05, **p < 0.01, ***p < 0.001 vs the saline group; ^#^*p* < 0.05, ^##^*p* < 0.01, ^###^*p* < 0.001 vs the ISO group; ^¶^*p* < 0.05, ^¶¶^*p* < 0.01, ^¶¶¶^*p* < 0.001 vs the ISO + Empty-SPION-treated group

A schematic outline of the 14 day ISO model and treatments evaluated is shown (Fig. 1A). Compared to measurements obtained from saline-treated control mice, ISO-injured mice had a ~ 27% increase in inflammatory cell infiltration (387 ± 11 cells/field; Fig. 1B and C) within the LV, which corresponded with a ~ onefold (~ 20 ± 2 cells per field; Fig. 1D) and ~ 5.7-fold (~ 10 ± 1 cells per field; Fig. 1E) increase in the number of TNF-α and IL-1β-expressing cells within the LV at day 14 post-injury (all *p* < 0.001 vs saline group). This ISO-induced increase in LV inflammation was equivalently and significantly abrogated by Pump-RLX (322 ± 14 cells/ ~ 11 ± 1 TNF-α-expressing cells/ ~ 3 ± 1 IL-β-expressing cells per field), i.p injected SPION-RLX (346 ± 8 cells/ ~ 10 ± 1 TNF-α-expressing cells/ ~ 4 ± 1 IL-β-expressing cells per field) or p.o administered SPION-RLX (347 ± 7 cells/ ~ 12 ± 1 TNF-α-expressing cells/ ~ 4 ± 1 IL-β-expressing cells per field), but not Empty-SPIONs after 7 days of treatment (Fig. 1B–E).

**Fig. 2:**
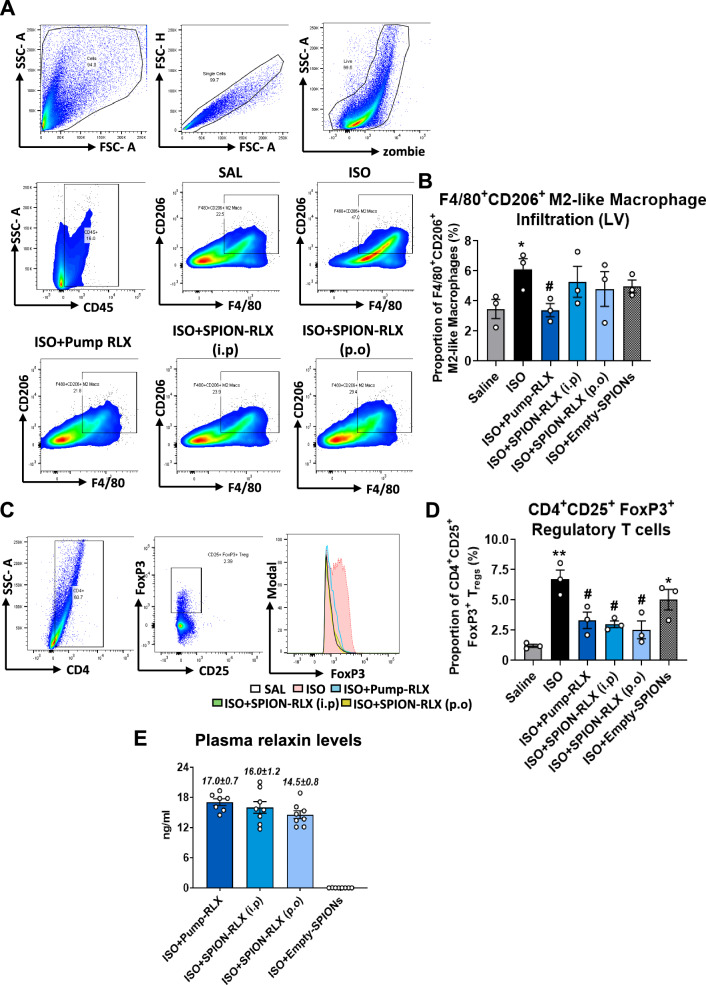
The effects of Pump-infused RLX vs i.p or drinking water (p.o)-delivered SPION-RLX on LV M2-like macrophage and regulatory T cell (T_reg_) infiltration in mice with cardiomyopathy, when administered from days 7–14 post-injury. **A**, **C** Representative FACS plots show the extent of **A** F4/80^+^ CD206^+^ positive M2-like macrophages and **C** CD4^+^ CD25^+^ FoxP3-positive T_regs_ in each of the groups indicated. **B**, **D** Also shown is the mean ± SEM proportion of **B** F4/80^+^ CD206^+^ positive M2-like macrophages or **D** CD4^+^ CD25^+^ FoxP3-positive T_regs_ in each of the groups shown. **E** Additionally shown is the mean ± SEM plasma (H2) relaxin (RLX) levels from mice subjected to repeated ISO administration, and treated with either Pump-infused RLX or i.p-or p.o-administered SPION-RLX post-ISO-injury) or i.p- or p.o-administered Empty-SPIONs, at day-14 post-injury. The data from panels **B** and **D** were obtained from n = 3 separate assays (tissue pooled from n = 2–3 mice per assay; from n = 7–8 mice per group); where as the data in panel **E** was obtained from n = 7–8 mice per group. The white coloured circles in each of the bar plots represent the individual data points per group. *p < 0.05, ** *p* < 0.01 vs the saline group; ^#^ *p* < 0.05vs the ISO group

Flow cytometry analysis of the LV revealed that ISO-injured mice had significantly reduced (by ~ 40–50%) infiltrating CD11c^+^ DCs (5.2 ± 0.7%; Fig. 1F and G) and CD11C^+^CD206^+^ monocyte-derived DCs (3.4 ± 0.6%; Fig. 1F and H), in comparison to measurements obtained from saline-treated controls (8.1 ± 0.6% and 6.7 ± 1.5%, respectively; Fig. 1G). Conversely, ISO-injured mice had significantly increased F4/80^+^CD206^+^ M2-like macrophage (6.1 ± 0.7%; Fig. 2A and B) and CD4^+^CD25^+^FoxP3^+^ regulatory T cell (T_reg_; 6.7 ± 0.8%; Fig. 2C and D) infiltration by day 14 post-injury compared to their saline-treated controls. Strikingly, RLX treatment, either through continuous pump administration or intermittent SPION-RLX administration, significantly promoted DC infiltration into the injured LV (by 2–3.5-fold for CD11c^+^ and CD11c^+^CD206^+^ DCs) after 7 days of treatment; an effect that was not induced by Empty-SPIONs alone (Fig. 1G and H). Furthermore, all forms of RLX treatment evaluated significantly ameliorated the ISO-induced increase in T_reg_ infiltration within the LV after 7 days (by ~ 60–75%; Fig. 2D); which again was not induced by Empty-SPIONs alone. On the other hand, only Pump-RLX abrogated the ISO-induced increase in LV F4/80^+^CD206^+^ M2-like macrophage infiltration within the LV (3.4 ± 0.7%; Fig. 2B).

Plasma (H2) RLX levels in mice treated with pump-RLX (17.0 ± 0.7 ng/ml) were found to be similar to that measured from mice intermittently treated with i.p-injected SPION-RLX (16.0 ± 1.2 ng/ml) or p.o-administered with SPION-RLX via daily drinking water consumption (14.5 ± 0.8 ng/ml) (Fig. 2E).

### Glycinated SPION-RLX was taken up by RXFP1-expressing DCs in the lungs of LPS-inflamed mice

**Fig. 3:**
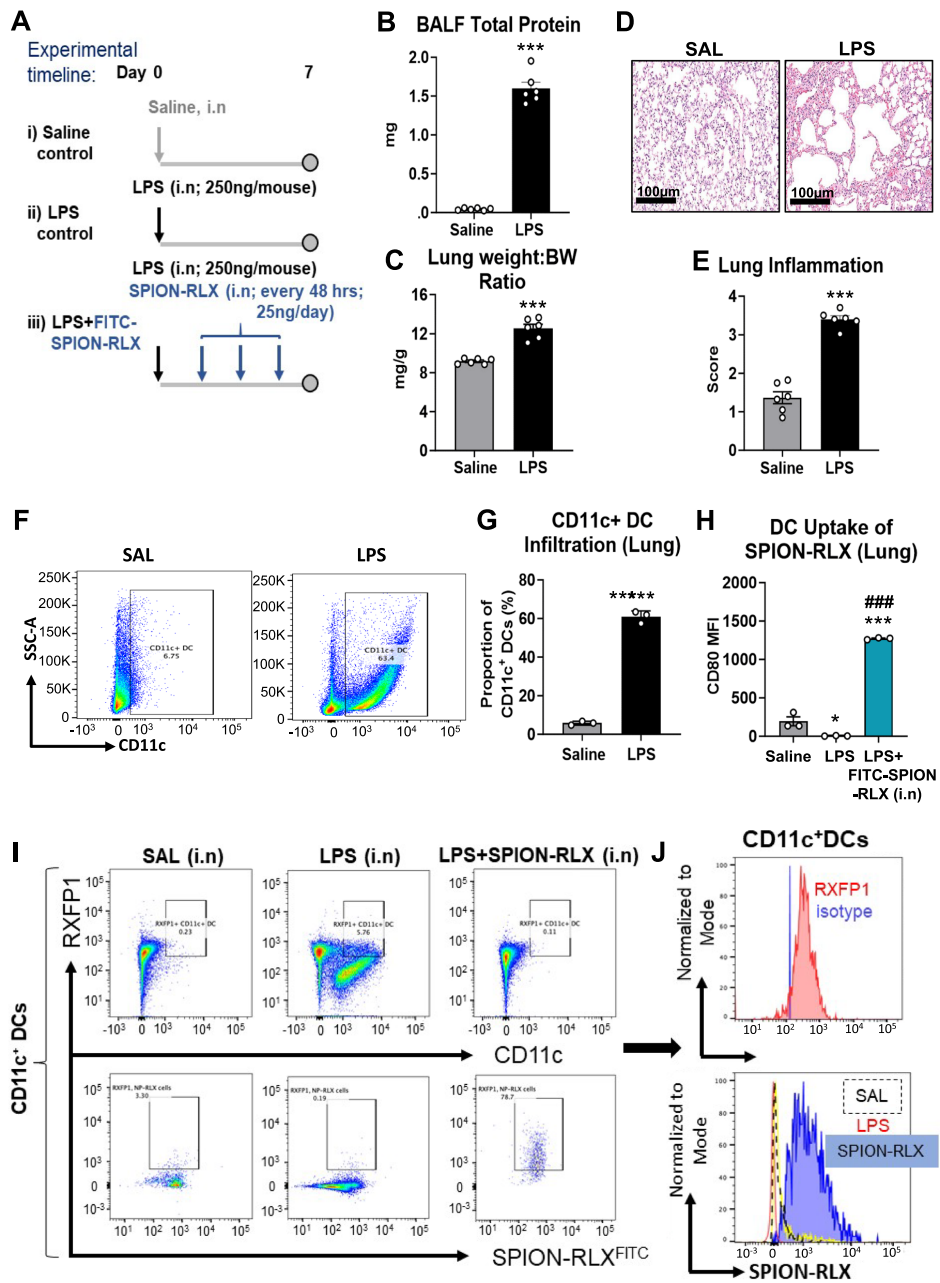
SPION-RLX was taken-up by infiltrating RXFP1-expressing dendrtic cells in the airways/lung of LPS-inflamed mice. **A** Schematic outline of the 7 day model of acute lung inflammation established and SPION-RLX^FITC^ treatment evaluated. **B**,** C** Shown is the mean ± SEM **B** total BALF protein and **C** lung weight:body weight (BW) ratio of LPS-inflamed mice at day 7 post-injury compared to their saline instilled counterparts. **D** Representative images of hematoxylin and eosin (H&E)-stained lung tissue sections show the extent of airway/lung inflammatory cell infiltration and widening of the alveolar space in LPS-inflamed mice compared to their saline instilled counterparts. **E** Also shown is the mean ± SEM number of inflammatory cells per field in LPS vs saline instilled mice. **F** Representative FACS plots show the extent of CD11c^+^ DCs within the lung of LPS at day 7 post-injury vs saline instilled mice. **G** Additionally shown are the mean ± SEM lung CD11c^+^ DCs from the FACS plots. **H** CD80 mean fluorescence intensity shows the DC uptake of SPION-RLX in LPS-inflamed mice. **I** Representative FACS plots show that infiltrating CD11c^+^ DCs within the lung expressed RXFP1 and were involved in the uptake of i.n administered FITC-labelled SPION-RLX. The data presented in panels **B**,** C** and **E** were obtained from n = 5–6 mice per group; whereas the data from panels **F**–**J** were obtained from n = 3 separate assays (tissue pooled from n = 2 mice per assay; from n = 6 mice per group). The white coloured circles in each of the bar plots represent the individual data points per group. *P < 0.05, ***P < 0.001 vs the saline group; ^###^*P* < 0.001 vs the LPS group

As systemically or orally delivered SPION-RLX significantly increased DC populations in the LV of ISO-injured mice (Fig. 1F–H), we next determined if CD11C^+^ DCs could take up i.n-administered SPION-RLX in LPS-inflamed mice with acute lung injury. A schematic outline of the 7 day model and treatments evaluated is shown (Fig. 3A). This model was chosen given that the inflammation induced by LPS enhances the uptake of nanoparticles by immune cells [34]. LPS-inflamed mice presented with significantly increased BALF total protein content (Fig. 3B), lung weight to body weight (BW) ratio (Fig. 3C) and lung inflammation (by ~ 1.5-fold; Fig. 3D and E) at 7 days post-injury, compared to respective measurements from saline-injected controls (all *p* < 0.001). CD11c^+^-gated DCs were ~ 9.5-fold higher (60.9 ± 1.3%) in the lung of LPS-instilled mice compared to respective measurements from saline-instilled controls (5.8 ± 0.4%) after 7 days (Fig. 3F and G). The T cell activating costimulatory molecule CD80 was used as an additional marker of activated cells. CD80 expression was decreased on DCs by ~ onefold of the mean fluorescence intensity (MFI) compared to respective measurements from saline-instilled controls after 7 days (Fig. 3H). On the other hand, CD80 MFI was markedly elevated by SPION-RLX^FITC^ treatment (by ~ sevenfold of levels measured in saline-instilled mice; Fig. 3H), suggesting that i.n administered SPION-RLX^FITC^ was predominantly taken up by these DCs.

The uptake of SPION-RLX^FITC^ by CD11c^+^ dendritic cells in LPS-instilled mice was confirmed by FACS analysis (Fig. 3I), which was ~ 75% greater than that observed in saline-instilled controls. The expression of RXFP1 was upregulated in DCs (Fig. 3J) following LPS-stimulation and treatment with SPION-RLX, compared to a concentration matched isotype control (FMO3), which gated for the negative population of RXFP1 absent cells and catered to any false-positive non-specific binding by the antibody isotype. This indicated that SPION-RLX was taken-up by RXFP1-expressing DCs.

### The systemic or oral delivery of glycinated SPION-RLX attenuated LV fibrosis, cardiomyocyte hypertrophy and vascular rarefaction in mice with cardiomyopathy

**Fig. 4:**
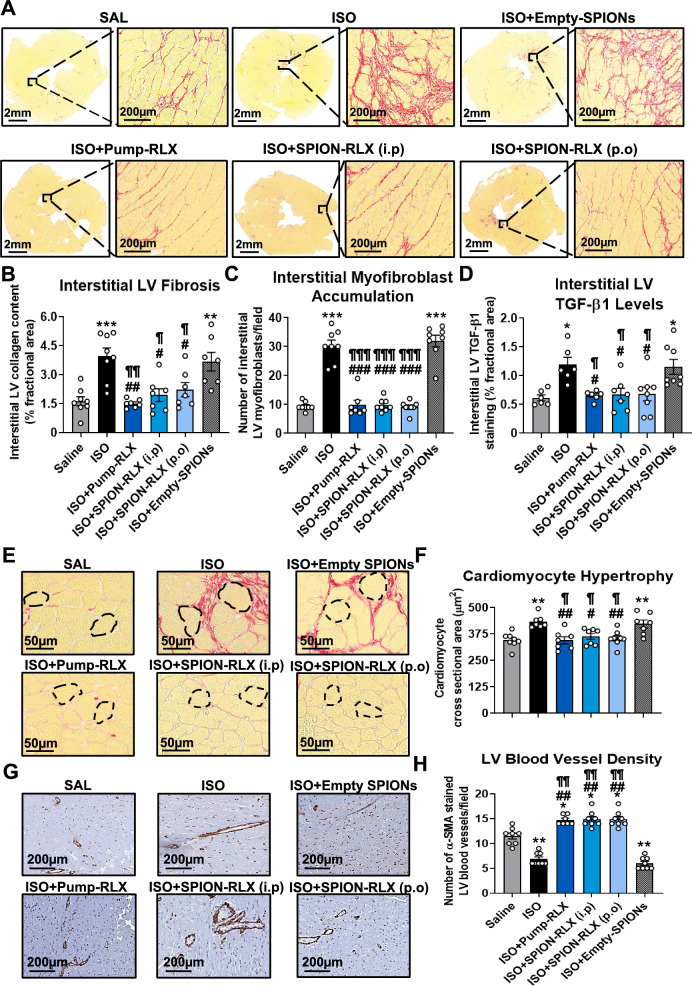
The effects of Pump-infused RLX vs i.p- or drinking water (p.o)-delivered SPION-RLX on measures of LV fibrosis, hypertrophy and vascular rarefaction in mice with cardiomyopathy, when administered from days 7–14 post-injury. **A** Representative images (with enlarged insets) of picrosirius red-stained LV tissue sections show the extent of interstitial collagen deposition within the LV midzone in each of the groups investigated. **B**–**D** Also shown are the mean ± SEM **B** % interstitial LV collagen deposition (fibrosis) per field, **C** number of interstitial myofibroblasts per field and **D** interstitial LV TGF-β1 levels per field from each group shown. **E** Representative picrosirius red-stained images also show the extent of LV cardiomyocyte size in each of the groups investigated. **F** The mean ± SEM LV cardiomyocyte cross sectional area in each of the groups analysed was then determined. **G** Representative immunohistochemically-stained sections for α-smooth muscle actin (SMA), show the extent of α-SMA-stained blood vessel density in each of the groups investigated. **H** Also shown is the mean ± SEM LV blood vessel density per group analysed. The data presented in panels **B**–**D**, **F** and **H** were obtained from n = 6–8 mice per group. The white coloured circles in each of the bar plots represent the individual data points per group. *p < 0.05, **p < 0.01, ***p < 0.001 vs the saline group; ^#^*p* < 0.05, ^##^*p* < 0.01, ^###^*p* < 0.001 vs the ISO group; ^¶^*p* < 0.05, ^¶¶^*p* < 0.01, ^¶¶¶^*p* < 0.001 vs the ISO + Empty-SPION-treated group

Repeated ISO administration to mice induced significantly increased (by ~ 1.5-fold) interstitial LV collagen deposition (fibrosis) at day-14 post-injury (4.0 ± 0.4%), compared to respective measurements from saline-injected controls (1.6 ± 0.2%; Fig. 4A and B). This LV fibrosis was accompanied by significantly increased interstitial LV myofibroblast accumulation (by ~ 2.2-fold; ~ 30 cells/field vs ~ 9 cells/field in saline-injected mice; Fig. 4C and Additional file 1: Fig. S3A) and TGF-β1 expression levels (by ~ onefold; 1.2 ± 0.1% vs 0.6 ± 0.1% in saline-injected mice; Fig. 4D and Additional file 1: Fig. S3B) at the time-point studied. Strikingly, the intermittent systemic (i.p) or oral administration of SPION-RLX abrogated the ISO-induced interstitial LV collagen fibrosis (to ~ 2.0 ± 0.3%; Fig. 4B), myofibroblast accumulation (to 9–10 cells/field; Fig. 4C) and TGF-β1 expression levels (to 0.7 ± 0.1%; Fig. 4D) to an equivalent extent as Pump-RLX treatment, and to levels that were no different to that measured in saline-injected control mice. However, these therapeutic effects of SPION-RLX could not be maintained by Empty-SPIONs alone.

Morphometric analysis of picrosirius red stained-LV sections revealed a significant increase in LV cardiomyocyte cross-sectional area in ISO-injured mice (by ~ 26%; 434 ± 12µm^2^) in comparison to measurements from saline-injected controls (345 ± 12µm^2^; Fig. 4E and F). This ISO-induced increase in cardiomyocyte cross-sectional area was abrogated by minipump-infused RLX (345 ± 17µm^2^) or by SPION-RLX treatment when administered either i.p (363 ± 14µm^2^) or via drinking water (354 ± 13µm^2^) (all by ~ 80–100%), but not by Empty-SPION treatment (422 ± 19µm^2^; Fig. 4F).

Cardiac hypertrophy is closely associated with vascular rarefaction within the myocardium [35]. Accordingly, there was a ~ 36% reduction in LV blood vessel density in ISO-injured mice (7 ± 0.4 vessels per field) in comparison to their saline-treated counterparts (11 ± 0.6; Fig. 4G and H). However, all three forms of RLX treatment, either through pump-RLX (15 ± 0.3 vessels per field) or SPION-RLX administered i.p (15 ± 1 vessels per field) or p.o (15 ± 1 vessels per field) equivalently restored this ISO-induced loss of blood vessel density (all by ~ onefold over the ISO alone group; Fig. 4H), to levels that were ~ 30% higher than that measured in saline-treated controls (all *p* < 0.05 vs saline group). These findings agreed with RLX’s well-documented angiogenic effects [25, 36, 37]. Conversely, the ISO-induced vascular rarefaction was unaffected by Empty-SPION treatment (6 ± 0.4 vessels per field; Fig. 4H).

### The anti-fibrotic effects of glycinated SPION-released RLX may have been mediated via an interaction between RXFP1 and scavenger receptors in the LV

**Fig. 5:**
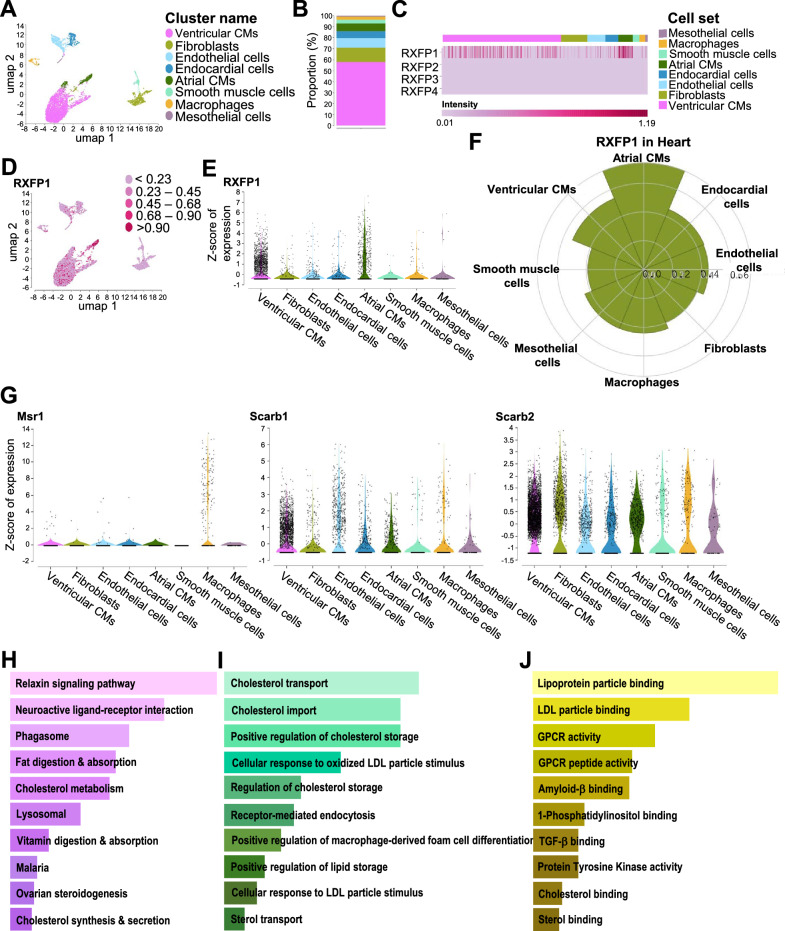
Map of the cellular composition of neonatal (P0) C57BL/6 mouse heart dataset from ParseBioscience. **A** A UMAP plot of scRNA-seq data from the C57BL/6 mouse heart identified 8 clusters of cells. **B** A stacked bar plot shows the relative cell proportion that was identified in the heart, expressed as a percentage. **C** The heatmap of *RXFP1, RXFP2, RXFP3* and *RXFP4* expression across all identified cell clusters. **D** A feature plot of *RXFP1* expression across all the UMAP identified clusters. **E** A violin plot depicting differential expression of *RXFP1* across the 8 different UMAP clusters. **F** Comparing the biased expression level in *RXFP1* expression by log^2^FC and –log10 (adjusted P value) demonstrated as a Rose plot. **G** Serial volcano plots of scavenger receptors that were identified across the different UMAP clusters. **H**–**J** The key target genes identified from Cellchat-analysed interactions between cardiomyocytes and macrophages, that focused on interactions between *RXFP1* and the scavenger receptors, *Msr1, Scarb1 and Scarb2,* using EnrichR are shown. Data shown were **H** sorted from high to low with a combined score of adjusted *p* value and FDR within the 2019 KEGG pathway (pink-shaded bars); **I** 2023 GO Biological processes (green-shaded bars); and **J** GO Molecular function (yellow-to-brown-shaded bars) databases. *LDL* low density lipoprotein, *GPCR* G protein-coupled receptor, *TGF* transforming growth factor

To elucidate potential mechanisms by which the RLX released from glycinated SPIONs may have been mediating its anti-fibrotic effects, scRNA-seq profiles from a C57BL/6 mouse heart dataset was generated from ParseBiosciences v1.0.3. A total of 9,243 cells with 44,635 mean reads/cell and 3,184 genes/cell that passed quality control filtering were retained for subsequent analysis. UMAP-clustering with Seurat v4.0 analysis identified 8 cell clusters, which were annotated using established marker genes (Fig. 5A and B). The major cell type in these clusters was ventricular cardiomyocytes (n = 4662), followed by fibroblasts (n = 1023), endothelial cells (n = 714), endocardial cells (n = 502), arterial cardiomyocytes (n = 566), smooth muscle cells (n = 269), macrophages (n = 228) and mesothelial cells (n = 81). Expression of RXFPs were investigated within the individual clusters. Interestingly, the cognate receptor for human gene-2 relaxin, *RXFP1* but not *RXFP2*, *RXFP3* or *RXFP4* (Additional file 1: Fig. S4A-C) was found to be predominantly expressed in atrial and ventricular cardiomyocytes (Fig. 5C-E) over its expression in macrophages and the other cell types identified (Fig. 5F). Relative to this, the expression of the scavenger receptors *Msr1, Scarb1* and *Scarb2* on these same cell clusters was also evaluated (Fig. 5G and Additional file 1: Fig. S4D). While *Msr1* was mainly expressed on macrophages, *Scarb1* and *Scarb2* were present in all identified clusters, albeit in differential proportions. Notably, *Scarb2* was also relatively highly expressed in atrial and ventricular cardiomyocytes, fibroblasts and macrophages (Fig. 5G). Using CellChat, an interaction between *RXFP1* and these scavenger receptors expressed on cardiomyocytes and macrophages was identified. The top-10 hit genes were further subjected to EnrichR analysis visualised here as a bar graph sorted by significant combined score of *p-*value and FDR (Fig. 5H-J). The results from the 2019 KEGG database demonstrated the implications of relaxin signalling, phagosomal activity and cholesterol metabolism as the major interactomes (Fig. 5H). Furthermore, analysis using the 2023 GO Biological Process database highlighted the possibility of cholesterol transportation for nanoparticle interactions and receptor mediated endocytosis (GO:0006898, Fig. 5I). A detailed investigation of the *RXFP1* and *Scarb2* interaction in cardiomyocytes, using the 2023 GO Molecular function database indicated the possibility of *TGF-β* binding being affected by the SPION interaction (GO: 0050431, Fig. 5J).

### The systemic or oral delivery of glycinated SPION-RLX promoted the balance between collagen-degrading MMPs and their TIMPs in the LV of mice with cardiomyopathy

**Fig. 6:**
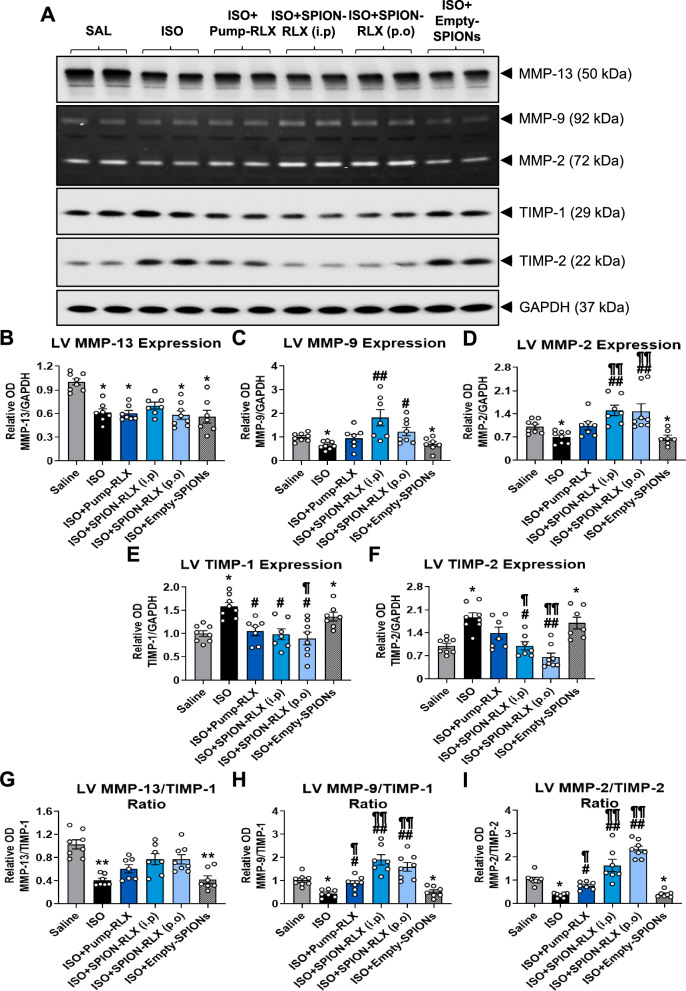
The effects of Pump-infused RLX vs i.p or drinking water (p.o)-delivered SPION-RLX on measures of ECM turnover: matrix metalloproteinases (MMPs) and tissue inhibitors of metalloproteinases (TIMPs) in mice with cardiomyopathy, when administered from days 7–14 post-injury. **A** Representative Western blots of MMP-13, TIMP-1 or TIMP-2; and gelatin zymographs of MMP-9 or MMP-2 show the LV expression levels of each MMP or TIMP evaluated in each of the groups investigated. The full blots are shown in Additional file 1: Fig. S5. **B-I** Also shown is the relative mean ± SEM optical density (OD) of LV **B** MMP-13, **C** MMP-9, **D** MMP-2, **E** TIMP-1, **F** TIMP-2, **G** MMP-13/TIMP-1 ratio, **H** MMP-9/TIMP-1 ratio and **I** MMP-2/TIMP-2 ratio in each the groups evaluated; in each case expressed as a relative value to the respective saline (SAL) control group value (which was expressed as 1 in each case). The data presented in panels **B**–**I** were obtained from n = 7–8 mice per group. The white coloured circles in each of the bar plots represent the individual data points per group. *p < 0.05, **p < 0.01 vs the saline group; ^#^*p* < 0.05, ^##^*p* < 0.01 vs the ISO group; ^¶^*p* < 0.05, ^¶¶^*p* < 0.01 vs the ISO + Empty-SPION-treated group

ISO-injured mice underwent a significant loss of collagen-degrading MMP-13 (Fig. 6A and B), MMP-9 (Fig. 6A and C) and MMP-2 (Fig. 6A and D) (all by ~ 30–40%) levels, but a significant increase in TIMP-1 (by ~ 60%; Fig. 6A and E) and TIMP-2 (by ~ 85–90%; Fig. 6A and F) levels within the LV by day 14 post-injury. As a result of this, ISO-injured mice presented with a significant reduction in LV MMP-13/TIMP-1 (by ~ 60%; Fig. 6G), MMP-9/TIMP-1 (by ~ 55%; Fig. 6H) and MMP-2/TIMP-2 (by ~ 65%; Fig. 6I) ratios at day 14 post-injury, in line with the increased LV fibrosis in these mice (Fig. 4B). Pump-RLX treatment restored the ISO-induced loss of LV MMP-9 (Fig. 6C) and MMP-2 (Fig. 6D) levels, significantly reduced the ISO-induced increase in LV TIMP-1 levels (Fig. 6E) and blunted the ISO-induced increase in LV TIMP-2 expression (Fig. 6F) to levels that were no longer different to that measured in saline-injected controls. This resulted in Pump-RLX treatment restoring the ISO-induced loss of LV MMP-13/TIMP-1, MMP-9/TIMP-1 and MMP-2/TIMP-2 ratios Fig. 6G–I) to levels that were equivalent to that measured in saline-injected control mice. On the other hand, i.p or p.o-administered SPION-RLX also significantly promoted LV MMP-9 (by ~ 1–1.80-fold over levels measured in ISO-injured mice; Fig. 6C) and MMP-2 (by ~ 1.1–1.2-fold over levels measured in ISO-injured mice; Fig. 6D) levels whilst being able to normalize the ISO-induced increase in TIMP-1 (Fig. 6E) and TIMP-2 (Fig. 6F). This resulted in SPION-RLX administration being able to restore the ISO-induced loss of LV MMP-13/TIMP-1 ratio (Fig. 6G) to levels measured in saline-injected control mice, but significantly increasing the LV MMP-9/TIMP-1 (Fig. 6H) and MMP-2/TIMP-2 (Fig. 6I) ratios (by ~ 3–fivefold over levels measured in ISO-injured mice). Hence, SPION-RLX promoted the balance between collagen-degrading MMPs and TIMPs that would facilitate the MMP-induced degradation of established ECM, independently of the administration route applied.

### The systemic or oral delivery of glycinated SPION-RLX attenuated LV dysfunction in mice with cardiomyopathy

Compared to measurements obtained from saline-treated control mice, ISO-injured mice underwent a significant reduction in ejection fraction (47.3 ± 1.5% vs 58.1 ± 3.5% in saline-injected control mice) but significant increase in end systolic volume (26.8 ± 1.3 vs 20.8 ± 1.8 µl) (Table 1). ISO-injured mice also presented with a trend towards an increased end-diastolic volume (53.6 ± 3.5 µl vs 46.1 ± 1.7 µl, isovolumetric contraction time (19.0 ± 2.0 ms vs 15.9 ± 0.9 ms) and isovolumetric relaxation time (22.1 ± 1.3 ms vs 18.6 ± 1.6 ms) (Table 1), indicating that they had stiffened hearts. The ISO-induced loss of ejection fraction, and increase in end systolic and diastolic volume were equivalently restored by Pump-RLX or SPION-RLX treatment independently of the administration route applied.

**Table 1:** The effects of Pump-RLX versus i.p- or p.o-delivered SPION-RLX on measures of LV function in mice with ISO-induced cardiomyopathy, when delivered from days 7–14 post-ISO injury

Parameter measured	SAL (n = 7)	ISO (n = 7)	ISO + Pump-RLX (n = 7)	ISO + NP- RLX (i.p) (n = 7)	ISO + NP-RLX (p.o) (n = 7)
End systolic LV mass (mg)	45.6 ± 3.7	58.8 ± 3.9	43.4 ± 3.6	42.1 ± 2.7	42.3 ± 3.1
End diastolic LV mass (mg)	44.3 ± 3.1	53.2 ± 2.6	41.5 ± 3.0	43.3 ± 2.1	43.7 ± 2.7
LVPWT (systole) (mm)	1.2 ± 0.1	1.2 ± 0.1	1.2 ± 0.1	1.2 ± 0.1	1.2 ± 0.1
LVPWT (diastole) (mm)	0.8 ± 0.1	0.8 ± 0.1	0.8 ± 0.1	0.8 ± 0.1	0.9 ± 0.1
Stroke volume (μL)	24.2 ± 2.4	27.9 ± 3.9	21.9 ± 1.8	24.2 ± 1.7	23.0 ± 1.1
Ejection fraction (%)	58.1 ± 3.5	47.3 ± 1.5*	56.2 ± 1.8^#^	56.0 ± 2.2^#^	56.9 ± 1.1^#^
Fractional Shortening (%)	25.6 ± 2.2	22.3 ± 1.4	29.0 ± 2.0	29.0 ± 1.8	27.5 ± 0.7
End systolic volume (μL)	20.8 ± 1.8	26.8 ± 1.3*	18.2 ± 1.8^##^	19.2 ± 1.6^#^	17.9 ± 1.1^##^
End diastolic volume (μL)	46.1 ± 1.7	53.6 ± 3.5	40.1 ± 3.3^#^	41.9 ± 2.8^#^	40.8 ± 1.3^#^
E/A wave ratio	1.5 ± 0.1	1.5 ± 0.1	1.6 ± 0.1	1.5 ± 0.1	1.4 ± 0.1
IVCT (ms)	15.9 ± 0.9	19.0 ± 2.0	21.5 ± 2.2	19.2 ± 1.1	22.7 ± 1.8
IVRT (ms)	18.6 ± 1.6	22.1 ± 1.3	20.0 ± 2.4	21.2 ± 0.1	16.5 ± 1.4

Cardiac functional parameters measured on day 14 post-ISO-injury using Transthoracic Echocardiography are expressed as the mean ± SEM, from saline (SAL)-treated controls, ISO-injured mice alone and ISO-injured mice treated with either pump-RLX or NP-RLX (administered i.p or p.o) from days 7 to 14 post-injury; from n = 7 mice per group. Data were analysed using a one-way ANOVA followed by Tukey’s multiple comparisons test.

*LV* left ventricle, *LVPWT (systole)* LV posterior wall thickness at systole, *i.p* intraperitoneal, *IVRT* isovolumetric relaxation time, *LVPWT (diastole)* LV posterior wall thickness at diastole, *RLX* relaxin, IVCT: isovolumetric contraction time, *ISO* isoprenaline, *NP* nanoparticles, *p.o* per os (oral administration)

*p < 0.05 vs saline group; ^#^*p* < 0.05, ^##^*p* < 0.01 vs ISO-alone group

### Repeated oral glycinated SPION administration did not induce any major adverse effects on animal mortality or organ pathology

**Fig. 7:**
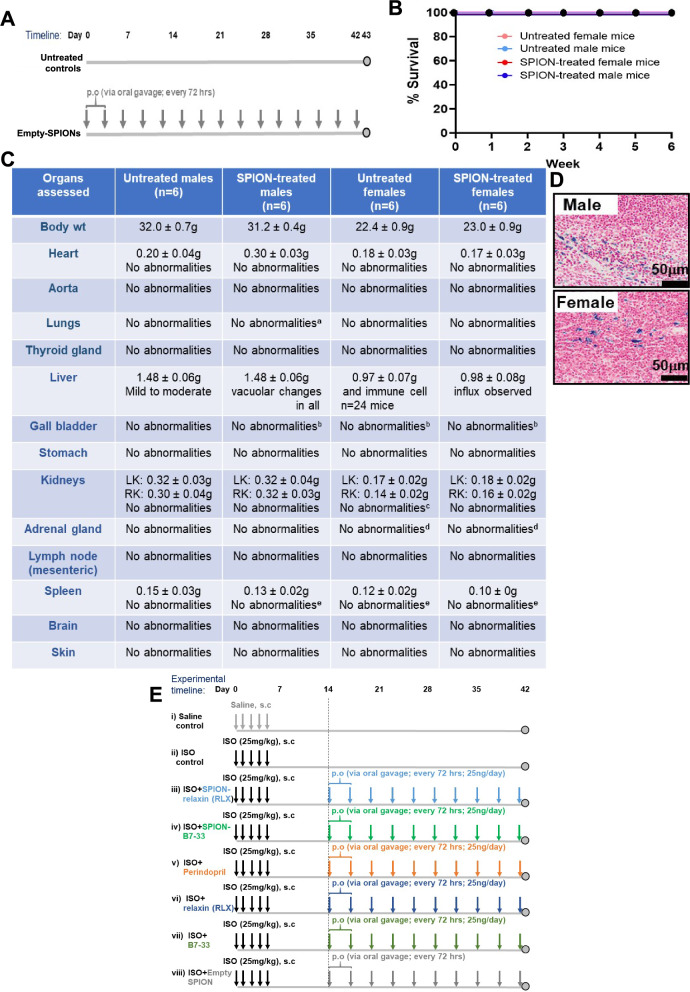
Repeated glycine-coated SPION administration did not induce any major adverse effects in mice. **A** Schematic outline of the safety study conducted in healthy mice, whereby male (n = 6/sex) or female (n = 6/sex) mice were left untreated or were repeatedly treated (15 times) with Empty-SPIONs over a 6 week period. **B** Repeated SPION administration did not affect animal mortality over the 6 week period. **C** A summary of the pathological assessment of the major organs isolated from untreated versus repeated SPION-treated healthy mice. ‘No abnormalities’ implies that no significant abnormalities were detected in these mice. However, mild to moderate vacuolar changes in immune cell influx were identified in all n = 24 untreated and SPION treated mice. Furthermore: ^a^3 out of 6 untreated female mice presented with mild pleural hemorrage with variable amounts of fibrin in their lungs; ^b^3 out of 6 SPION-treated males and 3 out of 6 untreated female/2 out of 6 SPION-treated females presented with mild epithelial hyperplasia in their gall bladder; ^c^1 out of 6 untreated female mice had mild to moderate hydronephrosis in their kidneys; ^d^3 out of 6 untreated females and 1 out of 6 SPION-treated females presented with cortical subcapsular spidle cell hyperplasia in their adrenal gland; ^e^4 out of 6 SPION-treated males and all untreated and SPION-treated females presented with mild to moderate focal diffuse lymphoid hyperplasia in their spleen. **E** Addtionally shown is the schematic outline of the 42 day model of cardiomyopathy established and treatments (timing, delivery routes, dosing) evaluated (for the data shown in Fig. 8)

A schematic outline of the 6 week safety study conducted is shown (Fig. 7A). Compared to healthy male and female adult mice that were untreated for 6 weeks, the repeated oral administration of glycinated Empty-SPION administration to mice over a 6 week period (every 72 h) did not affect animal mortality (Fig. 7B) or induce any major abnormalities in the heart, aorta, lungs, thyroid gland, liver, gall bladder, stomach, kidneys, adrenal gland, mesenteric lymph node, spleen, brain or skin of mice (Fig. 7C). Only mild to moderate vacuolar changes and immune cell influx was observed within the liver of all untreated and Empty-SPION treated male and female mice. These SPIONs were only detected in the spleen of male and female mice (Fig. 7D), which is where nanoparticles can end up before being cleared from the bloodstream. A schematic outline of the 42 day ISO model and treatments evaluated is also outlined (Fig. 7E).

### Repeated oral glycinated SPION-RLX or SPION-B7-33 delivery induced greater therapeutic efficacy compared to a frontline ACE inhibitor in mice with cardiomyopathy

**Fig. 8:**
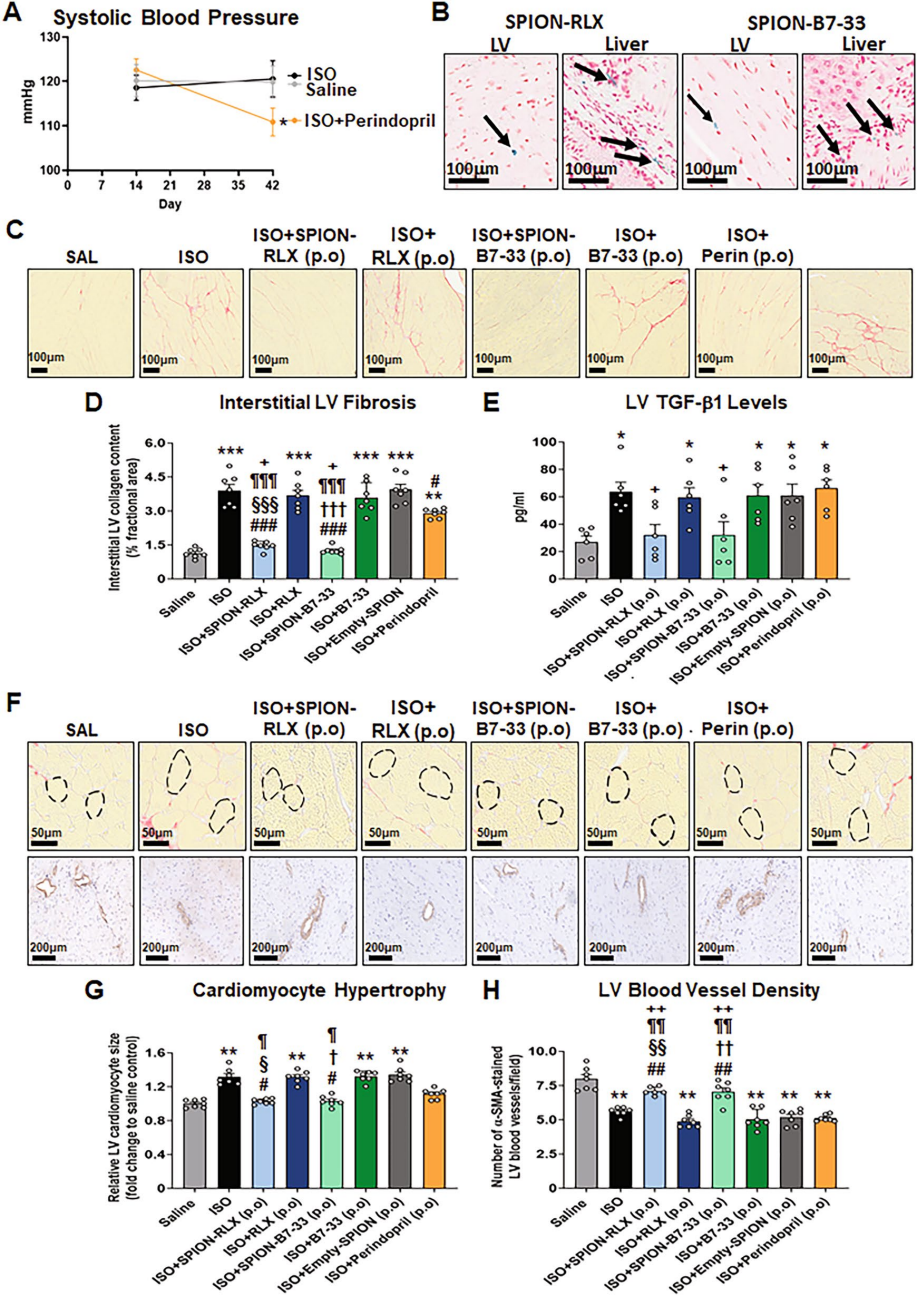
The longer-term effects of p.o delivered SPION-RLX or SPION-B7-33 vs the ACE inhibitor, perindopril, on measures of LV fibrosis, hypertrophy an vascular rarefaction in mice with cardiomyopathy, when administered from days 14–42 post-injury. **A** Shown is the mean ± SEM systolic blood pressure (SBP) for saline-injected, ISO-injected and ISO-injected + perindopril-treated mice (from n = 7 mice per group) at day 14 (prior to perindopril treatment) and day 42 (after 4-weeks of perindopril treatment). **B** Perl’s Prussian Blue staining of iron within the LV and liver of SPION-RLX- or B7-33-treated mice indicated that SPION-RLX or SPION-B7-33 was able to reach the target site (LV) and liver after 4-weeks of administration to ISO-injured mice. **C** Representative images of picrosirius red-stained LV tissue sections show the extent of interstitial collagen deposition within the LV midzone in each of the groups investigated. **D**–**F** Also shown are the mean ± SEM **D** %interstitial LV collagen deposition (fibrosis) per field and **E** LV TGF-β1 levels per field. **F** Representative picrosirius red-stained images also show the extent of LV cardiomyocyte size in each of the groups investigated. **G** The mean ± SEM LV cardiomyocyte cross sectional area in each of the groups analysed was then determined. **H** Representative immunohistochemically-stained sections for α-smooth muscle actin (SMA) show the extent of α-SMA-stained blood vessel density in each of the groups investigated. **I** Also shown is the mean ± SEM LV blood vessel density per group analysed. The data presented in panels **D**, **E**, **G** and **I** were obtained from n = 6–7 mice per group. The white coloured circles in each of the bar plots represent the individual data points per group. **p < 0.01, ***p < 0.001 vs the saline group; ^#^*p* < 0.05, ^##^*p* < 0.01, ^###^*p* < 0.001 vs the ISO group; ^§^*p* < 0.05, ^§§§^*p* < 0.001 vs the ISO + RLX-treated group; ^¶^*p* < 0.05, ^¶¶¶^*p* < 0.001 vs the ISO + Empty-SPION-treated group; ^+^*p* < 0.05 vs the ISO + Perindoril-treated group

The longer-term cardioprotective effects of p.o-administered SPION-RLX or its single-chain derivative, SPION-B7-33, were then compared to that of unconjugated RLX or B7-33 alone, Empty-SPIONs alone or perindopril alone. Perindopril treatment of normotensive ISO-injured mice reduced blood pressure (by 10–12 mmHg; Fig. 8A), moderately reduced the ISO-induced interstitial LV fibrosis (by ~ 35%; Fig. 8C and D) and normalised the ISO-induced cardiomyocyte hypertrophy (Fig. 8F and G) after 4 weeks of treatment, confirming that it was active at the dose administered. However, the ACE inhibitor failed to affect the ISO-induced LV TGF-β1 expression levels (Fig. 8E) and vascular rarefaction (Fig. 8F and H) over the same treatment period. Comparatively, p.o-administered SPION-RLX or SPION-B7-33 was identified in the LV (target site) and liver (the site at which nanoparticles reach before being cleared by the body) (Fig. 8B), and normalised the ISO-induced interstitial LV fibrosis (Fig. 8D), TGF-β1 expression levels (Fig. 8E), cardiomyocyte hypertrophy Fig. 8G) and vascular rarefaction (Fig. 8H) after 4 weeks of treatment. As a result of this, SPION-RLX or SPION-B7-33 significantly reduced the ISO-induced interstitial LV fibrosis (Fig. 8D), TGF-β1 expression levels (Fig. 8E) and vascular rarefaction (Fig. 8H) to a significantly greater extent than perindopril treatment. These effects of p.o-administered SPION-RLX or SPION-B7-33 were not maintained by orally administered RLX or B7-33 alone or Empty-SPIONs alone.

## Discussion

This is the first study to demonstrate that the conjugation of short-acting and poorly absorbed peptide therapeutics, recombinant human gene-2 relaxin (serelaxin; RLX) and its single chain-derivative, B7-33, to glycine-coated biodegradable SPIONs, was able to prolong their therapeutic activity and improve the oral applicability. While SPIONs had previously been explored as oral delivery vehicles [38], our study demonstrated that glycine-functionalized SPIONs served as a safe and unique delivery platform which could facilitate the systemic bioavailability of orally administered peptide conjugates, maintain their efficacy and prolong their therapeutic potential when repeatedly administered. RLX in particular had been well studied for its anti-fibrotic and other organ-protective effects in various pre-clinical models of disease [13, 14, 37, 39–43] and in patients ungergoing clinical trial evaluation [44–47], albeit needing to be continuously infused or daily injected to maintain its activity over weeks to months. Notably, RLX not only inhibited the impact of several pro-fibrotic factors, such as TGF-β1, connective tissue growth factor, angiotensin II and/or endothelin-1 on fibroblast to myofibroblast differentiation and myofibroblast-mediated ECM synthesis and deposition, it promoted the balance between ECM-degrading MMPs and their inhibitors (TIMPs) to promote the MMP-induced breakdown of established fibrosis [13, 14, 39, 40]. These therapeutic effects of RLX were all maintained by B7-33 [18, 19, 48, 49]. Strikingly, even the intermittent administration of SPION-RLX or SPION-B7-33 maintained organ-protection when administered to mice with dilated cardiomyopathy via daily drinking water (p.o) administration or via oral gavage or an i.p injection every 72 h.

Although only indirect evidence was provided, the findings of the studies conducted in LPS-inflammed mice suggested that SPION-RLX^FITC^ appeared to be taken up by infiltrating RXFP1-expressing DCs in acute stages of disease pathology. This was consistent with i.p or p.o administered SPION-RLX significantly increasing CD11c^+^ and CD11c^+^CD206^+^ DCs within the LV of ISO-injured mice. Previous studies had shown that orally-administered nanoparticles can be taken up by DCs [50], which then migrate via draining lymph nodes within the lymphatic system [51, 52] to the circulation and heart (and lung). It is also known that damage-associated signals from injured organs can attract nanomaterials to sites of damage from the circulation, which can vary depending on the hydrophobicity of the NPs involved [53] which for glycine-coated SPION-RLX, is stable in biological mediums for several months. Of relevance to the findings of this study, upon sensing inflammatory stimuli, DCs enter lymphpatic vessels and migrate to lymph nodes [54, 55]. Furthermore, hydrophobic [53] and negatively charged NPs are increasingly taken up by DCs, which may explain how the uptake of SPION-RLX by DCs may have allowed for RLX to be measured in the circulation of SPION-RLX-treated mice after being i.p or p.o administered. As SPIONs were intentionally designed to degrade and release the compounds they are conjugated within sites of damage, this would have allowed the RLX released from degraded SPIONs to exert its therapeutic effects within the injured myocardium. However, these findings remain to be conclusively demonstrated. Hence, further studies requiring the in vivo tracking of SPION-RLX within the lymphatic system following its oral administration are required.

Havin said that, i.p or p.o administered SPION-RLX significantly attenuated pro-fibrotic T_reg_ infiltration, LV inflammation and pro-inflammatory mediator (TNF-α, IL-1β) expression levels, cardiomyocyte hypertrophy, TGF-β1 expression, myofibroblast accumulation, interstitial collagen deposition (fibrosis) and related systolic dysfunction when administered to ISO-injured mice. Furthermore, i.p or p.o administered SPION-RLX maintained RLX’s ability to promote the balance between collagen-degrading MMPs over their natural inhibitors (TIMPs) in the injured LV. The anti-fibrotic and cardioprotective effects of p.o administered SPION-RLX were maintained by p.o delivered SPION-B7-33, but not by the p.o administered peptides alone or empty-SPIONs alone. Collectively, these findings demonstrated that glycine-functionalised SPIONs provided a novel vehicle that protected the conjugated peptide (up to 48–72 h prior to SPION degradation), and significantly enhanced the longer-term oral activity of RLX and B7-33, without exerting any therapeutics effects per se. This also allowed orally delivered SPION-RLX or SPION-B7-33 to exert greater anti-fibrotic and cardioprotective efficacy over the clinically-used ACE inhibitor, perindopril, after 4 weeks of treatment; effects that had previously been found when comparing minipump-infused RLX or B7-33 to the effects ACE inhibitors over shorter treatment periods [19, 24].

The carboxyl terminal of glycine-functionalised SPIONs, with exposed carboxyl (COOH) groups, were conjugated to the N-terminus of RLX [9] or B7-33 using carbodiimide chemistry without any linker associated cytotoxicity [56], which allowed for the RXFP1-binding domain of RLX and B7-33 to be exposed when conjugated to glycine-coated SPIONs. ~ 10.5 µg of RLX (from a starting concentration of 0.05 mg/ml) was measured following its conjugation to glycine-coated SPIONs (using the H2 RLX Quantikine® ELISA); which was then further diluted to provide mice with 125 ng in 5 ml of drinking water per day or 25 ng per 200 µl per oral gavage or i.p-administration. Importantly, these glycine-coated SPIONs retained a high proportion of RLX activity even when applied at diluted (ng) levels to preclinical models of disease owing to the protective effect of the glycine-coated SPIONs, which themselves had a concentration of 200 µg/ml. Our previous studies had found that ~ 80–90% of the RLX that was i.n-administered in the form of glycine-coated SPION-RLX (5 ng/day or 25 ng/day; instilled every 48 h) to mice with chronic AAD, could be detected in the circulation of treated mice after a 7 day treatment period [9]. Comparatively, the current study determined that ~ 5–6% of SPION-RLX that was p.o-administered via drinking water was dectected in the circulation of mice with dilated cardiomyopathy after a 7 day treatment period. Importantly though, the RLX levels detected from p.o administered SPION-RLX was similar to that produced by continuous Pump-RLX treatment, and were within the optimal therapeutic dose range of RLX that induces its cardioprotective effects in preclinical models and human patients [13, 14, 39, 40]. However, these RLX levels could potentially be adjusted (increased) to compensate for the fact that only around 60% of any compound will successfully be conjugated to glycine-coated SPIONs and/or the blood clearance of SPIONs. Moreover, the 6 kDa size of RLX and 3 kDa size of B7-33 offered less steric hindrance and more bioavailability of RLX and B7-33, respectively, due to glycine protecting the SPION core, unlike silane-linker particles with larger size which are less preferred (for uptake) by antigen-presenting cells.

We employed scRNA-seq analysis of a publicly available dataset to provide potential insights into the key cell clusters in the murine heart that expressed *RXFP1,* but not *RXFP2-4*. The available data found RXFP1 to be primarily expressed in atrial and ventricular cardiomycytes and macrophages in the C57BL/6 mouse heart, which is consistent with previous findings on RXFP1 expression in the CD1 mouse [57], Sprague–Dawley rat [58–60] and human [61] heart. Interestingly, GSEA analysis revealed significantly increased scavenger receptor genes in these cells which may explain a possible role for how these myeloid cells behave phagocytically and can take up glycine-coated SPIONs for delivery during the inflammatory response to tissue injury. Enrichment analysis highlighted that RLX signalling and activation of endocytic pathways may have been mediated via the interaction between RXFP1 and the scavenger receptors, Msr1, Scarb1 and Scarb2 expressed in these cells, which may have played a role in contributing to the up-take of SPION-RLX (and SPION-B7-33) for release into the circulation and sites of tissue damage [62]. These scavenger receptors are all expressed by DCs [63, 64] and play a role in contributing to the ability of DCs to recognise and engul pathogens and particles. Whilst these receptors don’t directly bind to TGF-β1, they can influence TGF-β1 binding and signalling by DCs to maintain immune responses and tissue homeostasis [65]. Hence, while it could be speculated that these scavenger receptors may play a role in the uptake of SPION-RLX, conclusive evidence of their involvement needs to be verified in future studies using receptor colocalization, knockdown or blockade (with scavenger receptor inhibitors such as fucoidan or poly-IC).

## Conclusion

In conclusion, this study demonstrated that glycine-functionalised SPIONs could act as a targeted and safe drug delivery vehicle that prolonged the activity of RXFP1-binding peptides (RLX and B7-33), regardless of their route of administraton (i.p, p.o, i.n [9]), and most notably, improved the oral applicability of these peptides. The uptake of glycine-functionalised SPIONs by myeloid cells such as DCs potentially allowed for SPION-RLX and SPION-B7-33 to circumnavigate the gut and enter the circulatory system via the lymphatic system. These SPIONs then also degraded within 72 h to release the peptides they were conjugated to within injured organs, for these peptides to exert their therapeutic effects. However, Empty-SPIONs themselves did not exert any therapeutic activity per se. These findings have tremendous implications for orally delivering the therapeutic application of peptide therapies in general, although may be impacted by the size, hydrophobic content and charge of the peptides involved, which to date have required invasive and repetitive frequency of administration to maintain activity. The clinical translation of these findings will require scalable, GMP-compliant nanoparticle synthesis and careful assessment of long-term safety, particularly regarding potential pro-inflammatory effects of SPIONs which we have overcome by functionalising SPIONs with glycine.

## Supplementary Information

Additional file 1 (i) Supplementary Fig. 1: A schematic illustration of how the N-terminus of RLX or B7-33 were conjugated to SPIONs using carbodiimide chemistry; Supplementary Fig. 2: Characterisation of SPION-RLX and SPION-RLX^FITC^; Supplementary Fig. 3: Representative images of α-SMA and TGF-β1-stained LV sections from saline, ISO-injured and ISO-injured mice treated with minipump (Pump)-infused relaxin (RLX), i.p-administered SPION-RLX or drinking water (p.o)-administered SPION-RLX, from days 7-14 post-injury; (ii) Supplementary Fig. 4: Map of the cellular composition of the C57BL/6 mouse heart and CD1 mouse lung datasets from ParseBioscience; (iii) Supplementary Fig. 5: The full Western blots and gelatin zymographs that were cropped to create Fig. 5A.

## Data Availability

The data supporting the findings from this study are available within the manuscript. Any remaining raw data are archived in a Monash University repository and will be available from the corresponding author upon reasonable request.
