# Supplementary material for: Immune cell uptake of glycinated nanoparticles conjugated to anti-fibrotic peptides enables their prolonged activity and oral administration
Source: J Biomed Sci. 2025 Dec 12;32:104. doi: 10.1186/s12929-025-01198-8 (PMC12699924; doi:10.1186/s12929-025-01198-8)
Supplement: Supplementary file 1 — Additional file 1 (i) Supplementary Fig. 1: A schematic illustration of how the N-terminus of RLX or B7-33 were conjugated to SPIONs using carbodiimide chemistry; Supplementary Fig. 2: Characterisation of SPION-RLX and SPION-RLXFITC; Supplementary Fig. 3: Representative images of α-SMA and TGF-β1-stained LV sections from saline, ISO-injured and ISO-injured mice treated with minipump (Pump)-infused relaxin (RLX), i.p-administered SPION-RLX or drinking water (p.o)-administered SPION-RLX, from days 7-14 post-injury; (ii) Supplementary Fig. 4: Map of the cellular composition of the C57BL/6 mouse heart and CD1 mouse lung datasets from ParseBioscience; (iii) Supplementary Fig. 5: The full Western blots and gelatin zymographs that were cropped to create Fig. 5A. [file 12929_2025_1198_MOESM1_ESM.docx]

**Supplementary Information**

**
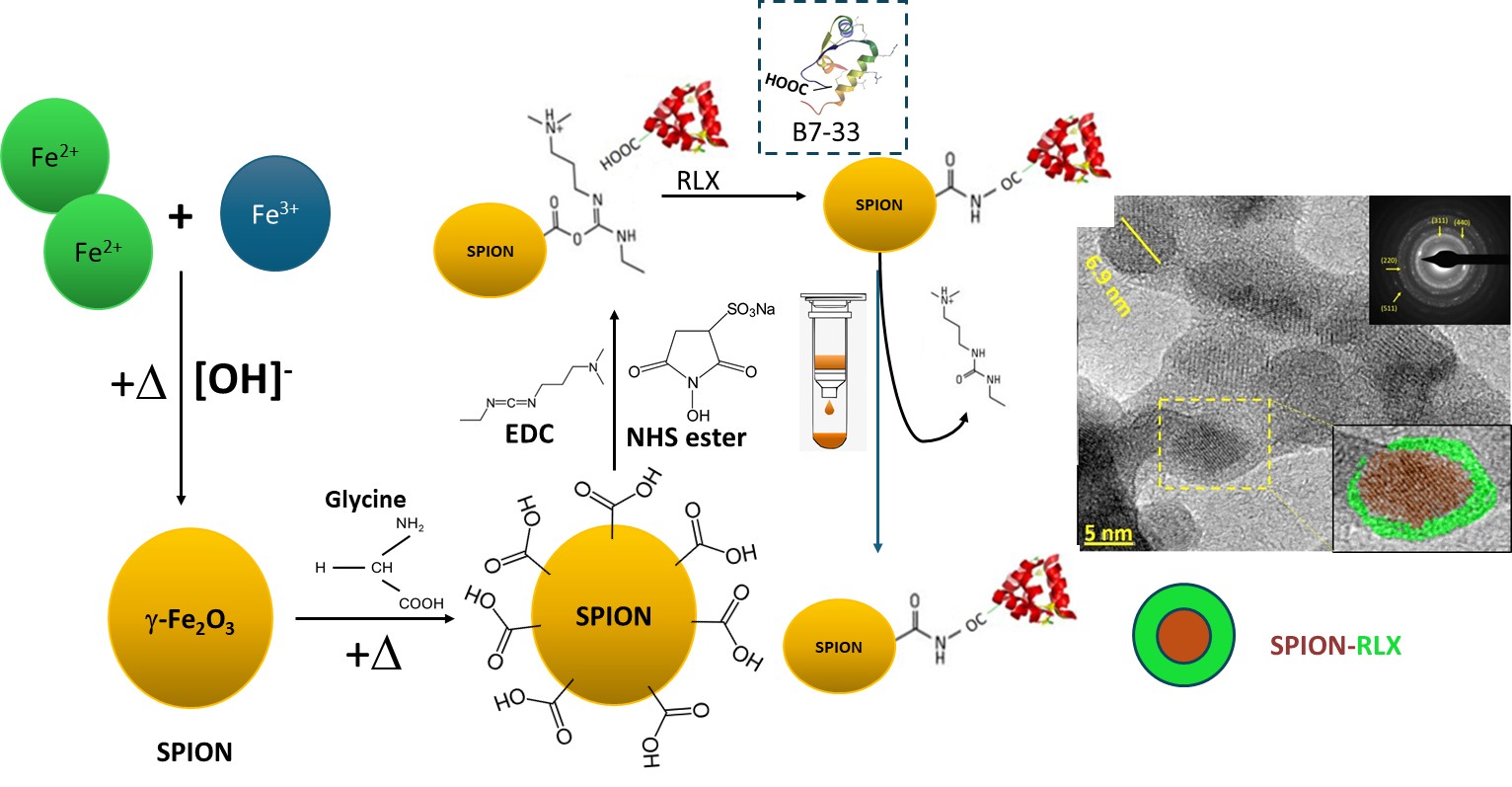
**

**Supplementary Fig. 1.** Schematic diagram of how the N-terminus of RLX or B7-33 were conjugated to carboxyl modified iron oxide nanoparticles (SPIONs) using carbodiimide chemistry, which were then glycine functionalised to dampen the immune response to SPION-RXFP1 ligand administration. Confirmation that SPIONs were successfully conjugated to RLX was previously demonstrated in [9].

**
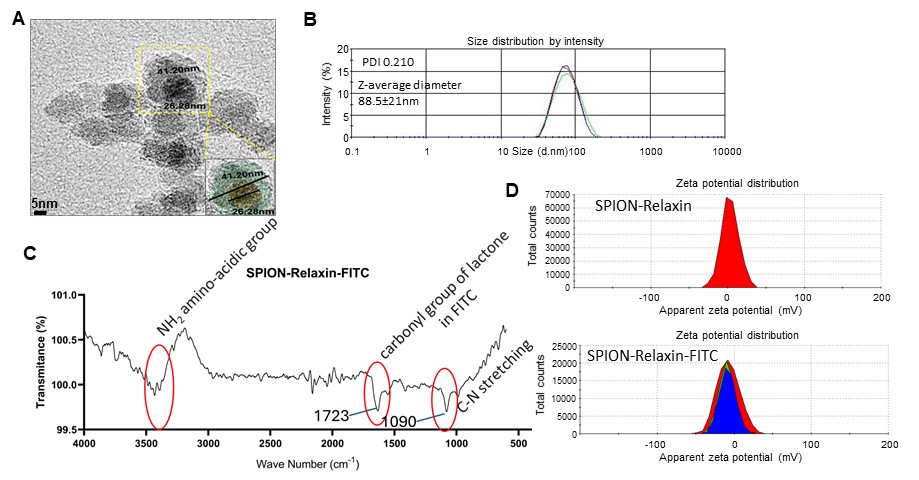
**

**Supplementary Fig. 2.** **Characterisation of SPION-RLX and NP-RLX^FITC^. A**TEM imaging of SPION-RLX with inset: pseudo-colour imaging of the SPION core in yellow (diameter 26.28 nm) and RLX as a corona in green (diameter 41.20 nm). **B**Hydrodynamic size of SPION-RLX with a poly-dispersity index (PDI) of 0.210 and average hydrodynamic diameter of 88.5 nm. **C**FTIR imaging of the SPION-RLX^FITC^ conjugation shows the NH_2_ terminal RLX peak at 3248 cm^-1^ and carbonyl group of lactone ring in FITC at 1723 cm^-1^. **D**Zeta-potential of SPION-RLX (-2.75mV) shown in red and zeta potential of NP-RLX^FITC^ conjugation (-7.45mV).


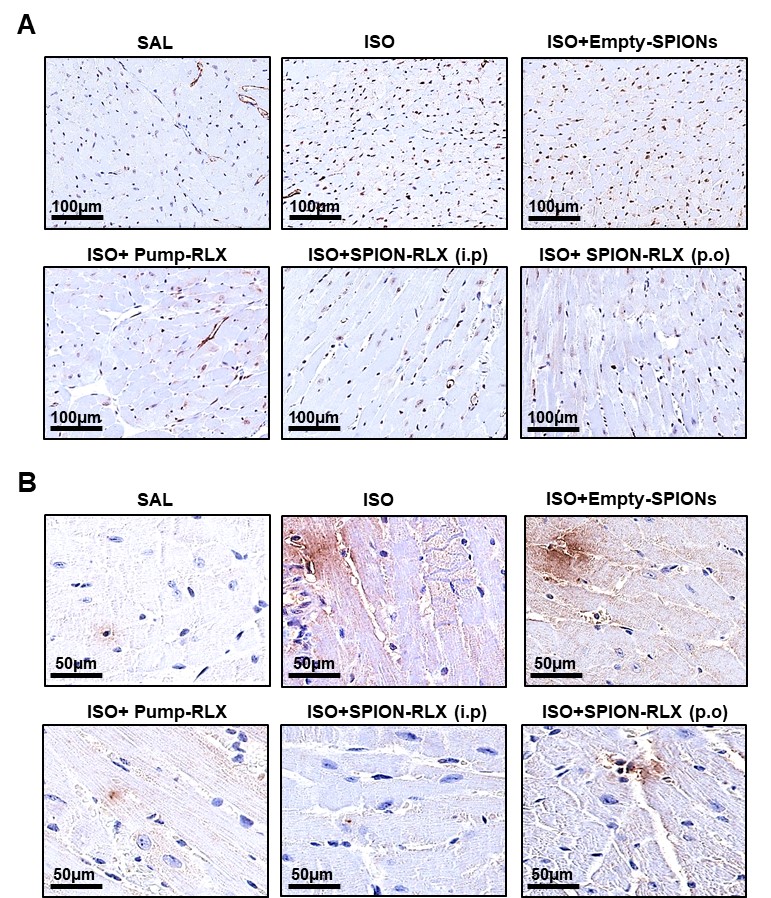


**Supplementary Fig. 3.** Representative images of α-SMA and TGF-β1-stained LV sections from short-ter (14 day) ISO model established. **A,B** Representative images of immunohistochemically-stained LV tissue sections show the extent (brown staining) of **A** interstitial α-SMA-stained myofibroblast density or **B** TGF-β1 expression in each the groups investigated. The morphometric analysis of this staining is presented in Fig. 3C and Fig. 3D, respectively.


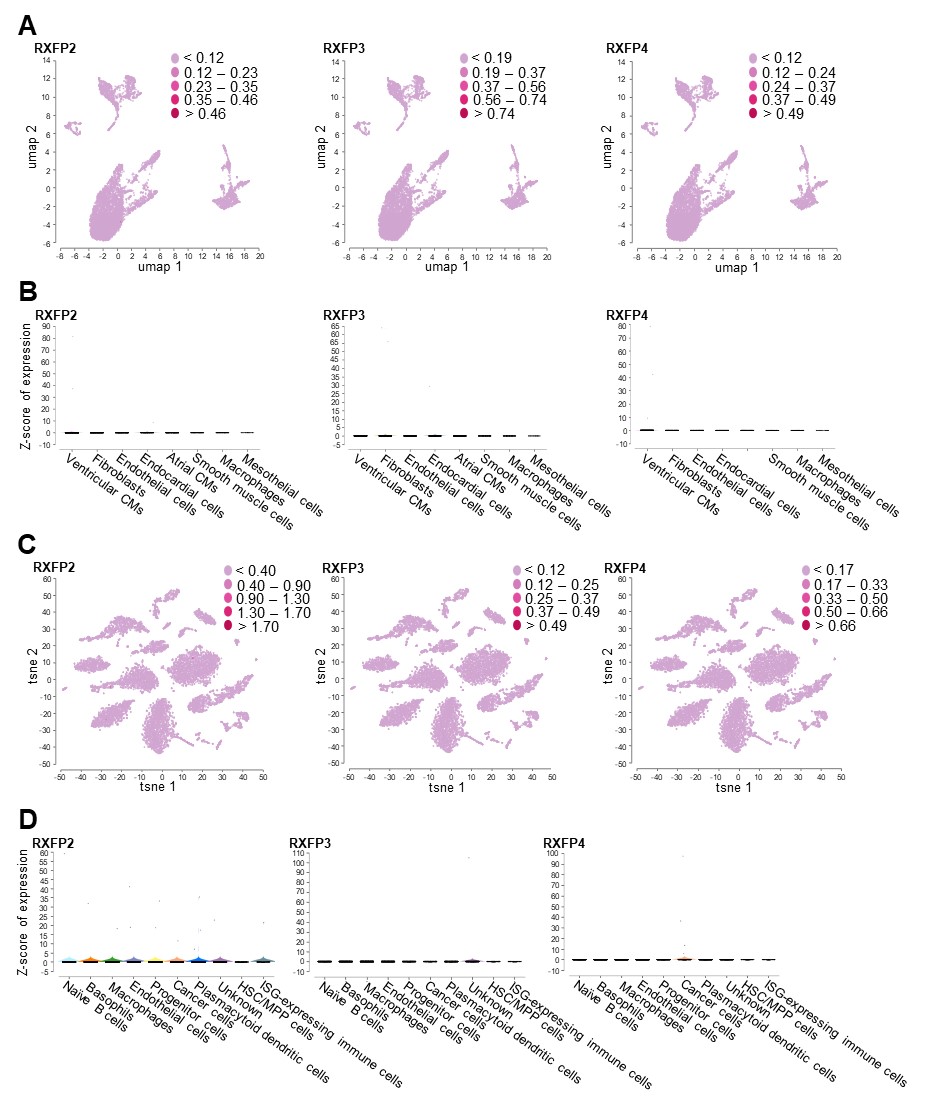


**Supplementary Fig. 4:** Map of the cellular composition of C57BL/6 mouse heart and CD1 mouse lung dataset from ParseBioscience. **A** Feature plot of *RXFP2*, *RXFP3* and *RXFP4* expression across all the UMAP identified cell clusters. **B** Serial volcano plots show the expression levels of *RXFP2, RXFP3 and RXFP4* across the different UMAP cell clusters in the neonatal heart dataset. **C** Feature plot of *RXFP2, RXFP3 and RXFP4* expression across all the tSNE-identified cell clusters. **D** Serial volcano plots show the expression levels of *RXFP2, RXFP3 and RXFP4* across the different tSNE cell clusters in the adult lung dataset.


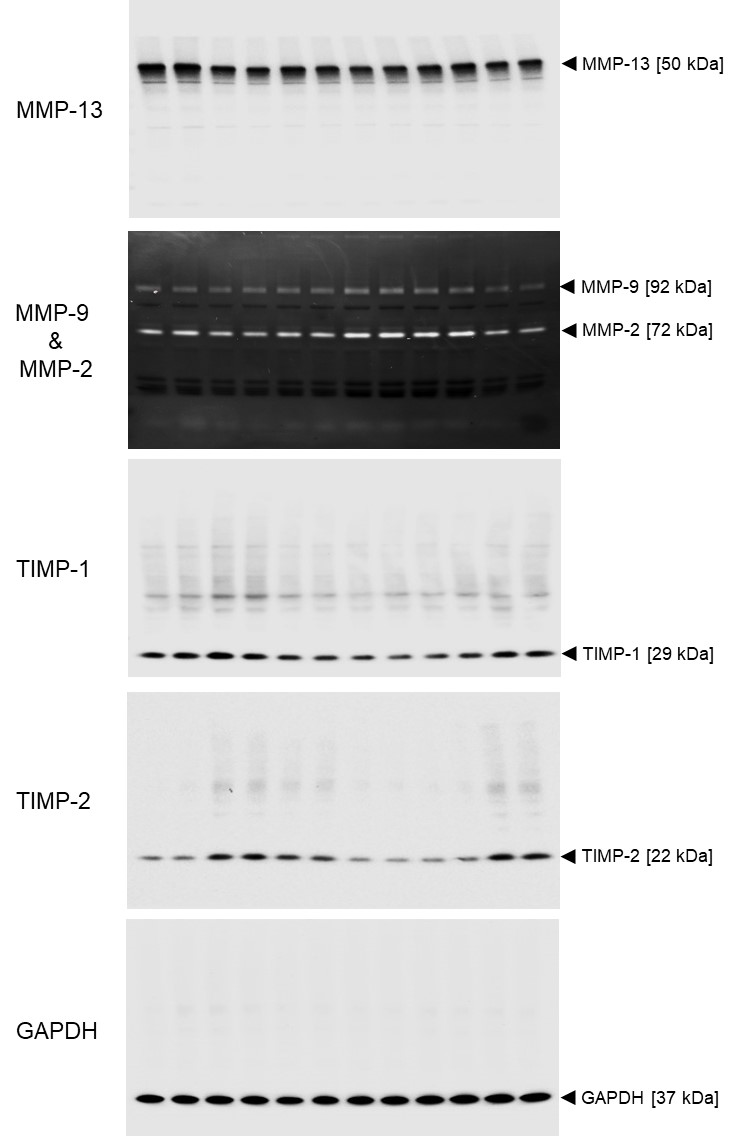


**Supplementary Fig. 5:** The full Western blots and gelatin zymographs that were cropped to create Fig. 6A.
